# Low-cost robotic manipulation of live microtissues for cancer drug testing

**DOI:** 10.1126/sciadv.ads1631

**Published:** 2025-05-14

**Authors:** Ivan Stepanov, Noah R. Gottshall, Alireza Ahmadianyazdi, Daksh Sinha, Ethan J. Lockhart, Tran N. H. Nguyen, Sarmad Hassan, Lisa F. Horowitz, Raymond S. Yeung, Taranjit S. Gujral, Albert Folch

**Affiliations:** ^1^ Department of Bioengineering, University of Washington, Seattle, WA, USA.; ^2^ Department of Mechanical Engineering, University of Washington, Seattle, WA, USA.; ^3^ Department of Surgery, University of Washington, Seattle, WA, USA.; ^4^ Human Biology Division, Fred Hutchinson Cancer Center, Seattle, WA, USA.

## Abstract

The scarcity of human biopsies available for drug testing is a paramount challenge for developing therapeutics, disease models, and personalized treatments. Microtechnologies that combine the microscale manipulation of tissues and fluids offer the exciting possibility of miniaturizing both disease models and drug testing workflows on scarce human biopsies. Unfortunately, these technologies presently require microfluidic devices or robotic dispensers that are not widely accessible. We have rapidly prototyped an inexpensive platform based on an off-the-shelf robot that can microfluidically manipulate live microtissues into/out of culture plates without using complicated accessories such as microscopes or pneumatic controllers. The robot integrates complex functions with a simple, cost-effective, and compact construction, allowing placement inside a tissue culture hood for sterile workflows. We demonstrated a proof-of-concept cancer drug evaluation workflow of potential clinical utility using patient tumor biopsies with multiple drugs on 384-well plates. Our user-friendly, low-cost platform promises to make drug testing of microtissues broadly accessible to pharmaceutical, clinical, and biological laboratories.

## Teaser

A low-cost robotic platform handles microtumors and catalyzes their use in cancer drug evaluation and personalized oncology.

## INTRODUCTION

The process of drug development is vastly inefficient (*1*, *2*). The final stage of drug screening—a “drug evaluation” where the top drug candidates are tested for efficacy and safety to decide which candidate is pushed onto a clinical trial—is especially critical. The traditional disease models based on tissue cultures and animals are extremely poor predictors of human disease outcomes, as shown by decades of clinical trials (*3*–*7*). In oncology, where the first line of treatment is often surgery, an obvious solution to the poor predictivity of animal testing is to directly test drugs ex vivo on human tissue fragments or constructs. However, the typical size of a tumor biopsy extracted during surgery is only ~1 cm^3^ (*8*), which presents challenges for testing multiple drugs. Furthermore, the rise of combination therapies (*9*) and dynamic therapies (*10*, *11*) are exponentially increasing the complexity and cost of testing, and the improvement in early detection techniques (*12*) keeps reducing the biopsy sizes obtained at the time of intervention.

As a result, there has been a fast-rising interest in miniaturizing the drug development process via the use of submillimeter-sized three-dimensional (3D) live tissues (“microtissues”). These microtissues enable inexpensive, more efficient tests of high clinical biomimicry that address microscale phenomena [such as diffusive mass transport and the role of tissue heterogeneity on therapeutic efficacy (*13*)] while maximizing the use of scarce materials (i.e., live biopsies) (*14*–*26*) and minimizing animal testing. The microtissues are produced either by bottom-up or top-down approaches. Bottom-up approaches include constructs such as organs-on-chips or organoids built by bioprinting (*27*, *28*), microengineering (*29*–*31*), or aggregation from single cells (*32*–*35*), often using cell growth to amplify the tissue. Conversely, the top-down approach consists of fragmenting biopsies by microdissection (without any growth), encompassing microdissected tissues such as organospheres, spheroids, and tumoroids (*15*–*26*). Microtissue-based drug tests are used not only for drug screening (*19*, *20*, *35*–*37*) but, increasingly, also for disease modeling in cancer (*15*, *38*) and immunology (*39*), for regenerative medicine (*40*), and for personalized medicine (*18*, *37*, *41*).

Because of the microtissues’ small size, their high-throughput manipulation and culture has spurred the development of high-precision microfluidic, bioprinting, and robotic platforms (*42*). Microfluidic tools such as hanging drops (*43*–*45*), droplet microfluidics (*18*, *28*), and/or microfluidic perfusion (*11*, *15*, *19*, *20*, *24*, *34*, *46*–*49*) ensure the fluidic compartmentalization and microenvironmental control of the microtissues; however, they entail complex fluid control systems that are not plug-and-play. Furthermore, microfluidic fabrication and operation require highly specialized human expertise, which can hamper their wider adoption in research settings and the translation of microtissues to the clinic. Various robotic platforms address the lack of user-friendliness of microfluidic platforms by automating the handling (i.e., pipetting and transferring) of microtissues and fluids. However, present robotic manipulators often target high-resolution manipulation of single cells requiring a microscope, a pneumatic controller, or both (*50*), and existing commercial systems typically integrate filtered air for sterility, resulting in bulky, expensive robots (see table S1).

**Fig. 1.**
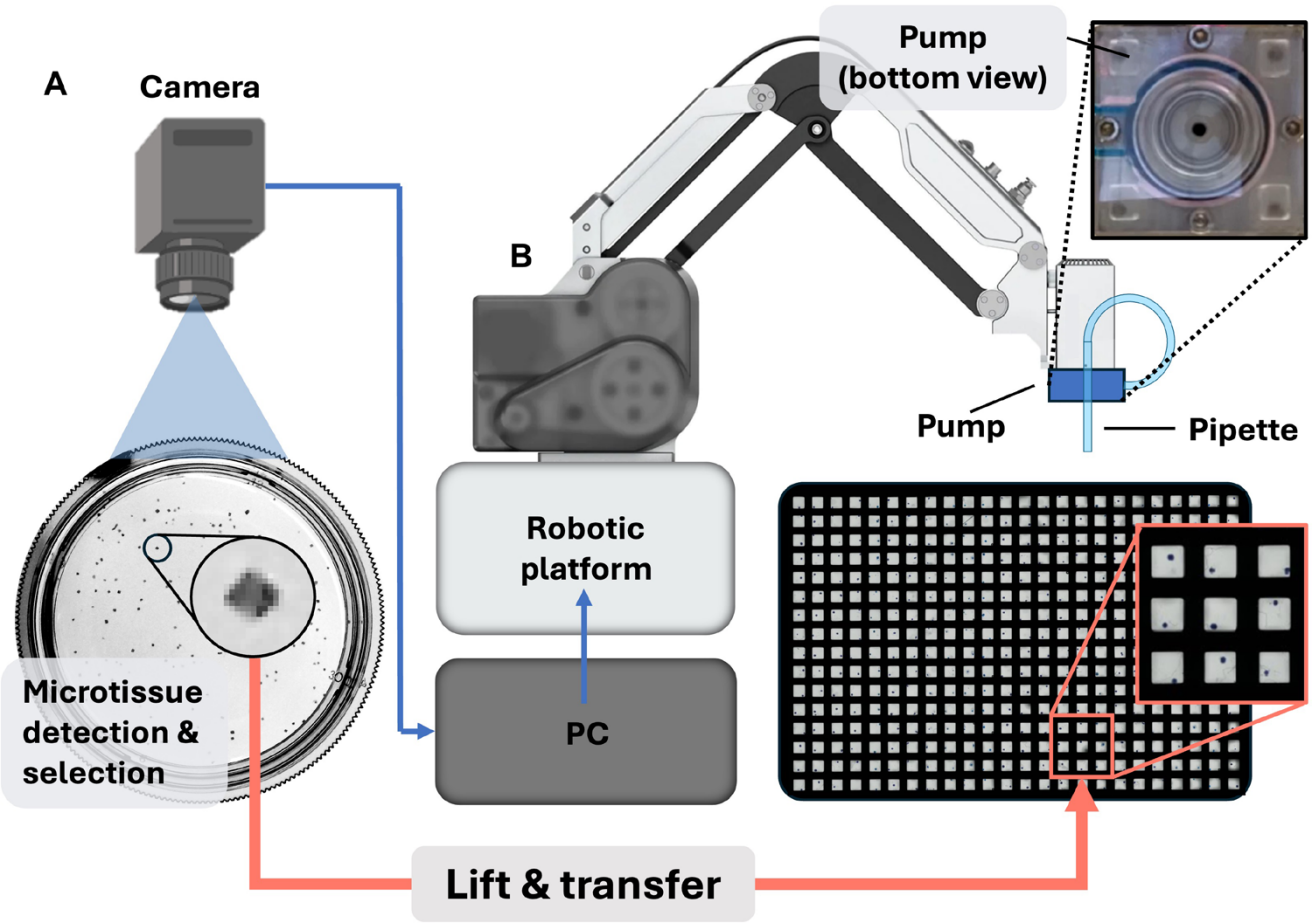
Robotic platform for automated “lift”-and-transfer of microtissues from a culture dish to a multiwell plate. (**A**) Schematic of the setup depicting the culture dish, a random distribution of microtissues (cuboids), and the USB camera above. Inset: Magnified view of a 400-μm-wide cuboid illustrating that the resolution of the USB camera is sufficient to detect cuboids. (**B**) Conceptual rendering of the robot’s operation depicting the image that guides the robot’s movements, the robot assembled with the pump, and the capillary at its head above a 384-well plate (inset: 3 × 3 array of wells containing 400-μm-wide microtissues). The microtissue distribution is analyzed by a PC using images taken by the camera. The software selects a suitable microtissue to be transferred to the target well plate by the robotic platform. Activating the pump, the robot “lifts” the microtissue into the capillary and transfers it to the target well. Top inset: Bottom view of the rotary pump.

To broaden access to microtissue research, we have rapidly prototyped a user-friendly, cost-effective, and compact robotic platform that enables the automated manipulation of microtissues in a standard tissue culture hood (Fig. 1 and fig. S1). Compared to existing commercial robotic dispenser systems, our system features three key advantages: (i) Instead of a microscope, a high-resolution USB camera (Fig. 1A) enables low-cost sorting and transfer of live microtissues. Aided by computer vision, the robot sequentially picks and sorts microtissues from a random distribution of microtissues in a culture dish into a multiwell plate (or any user-programmed array) (Fig. 1B). (We typically use nonadherent multiwell plates that result in slight motions of the microtissues inside the wells that have no effect on the analysis, but for other applications, adherent surfaces may be desirable.) (ii) Our platform builds on a compact off-the-shelf robot, a design that is compatible with standard sterile workflows (fig. S2). Robotic placement of the microtissues into a multiwell plate in the hood is followed by culture in a standard incubator. (iii) Fluid motion to manipulate tissues does not require a separate pneumatic controller or syringe pump; instead, the robot integrates a custom rotary pump powered by the motor in the end-effector (“head”) of the robotic arm (Fig. 1B, inset and Rotary pump in Supplementary Text). This pump generates the microfluidic flow needed to “lift” and dispense microtissues via a standard glass capillary. Thus, a single Python interface controls the pump via the translational motors, as well as the rest of the actions and user interface for the system. Hence, this integrated solution simplifies and substantially lowers the cost of programmable fluid manipulation.

To demonstrate the utility of our platform, we present robotic manipulations that enable facile drug testing of monodisperse microdissected tissues (both mouse and human) in 348-well plates. These “cuboids” result from three orthogonal cuts with a tissue chopper (see Materials and Methods), featuring a relatively uniform distribution of sizes and cuboidal shapes at day 0 (*19*, *46*), and retain the tumor microenvironment (TME) (*51*). Although shape uniformity is convenient, and the TME can play a critical role in drug efficacy, other types and shapes of microtissues such as minced tissues or organoids can also be used, and our freely available graphic user interface (GUI) permits size selection. Human tumor cuboids usually retain their cuboidal shape for a few days in culture, whereas mouse tumor cuboids rapidly evolve to a spheroid shape in the same period; hence, here we refer to the latter as “spheroidal cuboids” to avoid confusion, whereas the term “microdissected tissue” or “microtissue” applies to both. We show that the technology applies equally well for manipulating the tested microtissue shapes, sizes, and species. We demonstrate robotic protocols to pick and place single cuboids and sort hundreds of cuboids according to their size in separate wells in less than 1 hour. Last, we perform drug evaluations of cuboids with multiple drugs on 384-well plates, including the evaluation of clinically relevant drugs on a patient biopsy.

## RESULTS

### Microtissue pick-and-place using a microfluidic “lift”-and-transfer process

Operation of the robotic platform is largely automated through a GUI (see Materials and Methods). Through the GUI, the user calibrates the robot’s coordinate system (see Computer vision in Supplementary Text and fig. S3, A and B), programs the destination positions for the microtissues (such as a multiwell plate), and specifies the range of microtissue sizes to pick from (see Computer vision in Supplementary Text and fig. S3C).

**Fig. 2.**
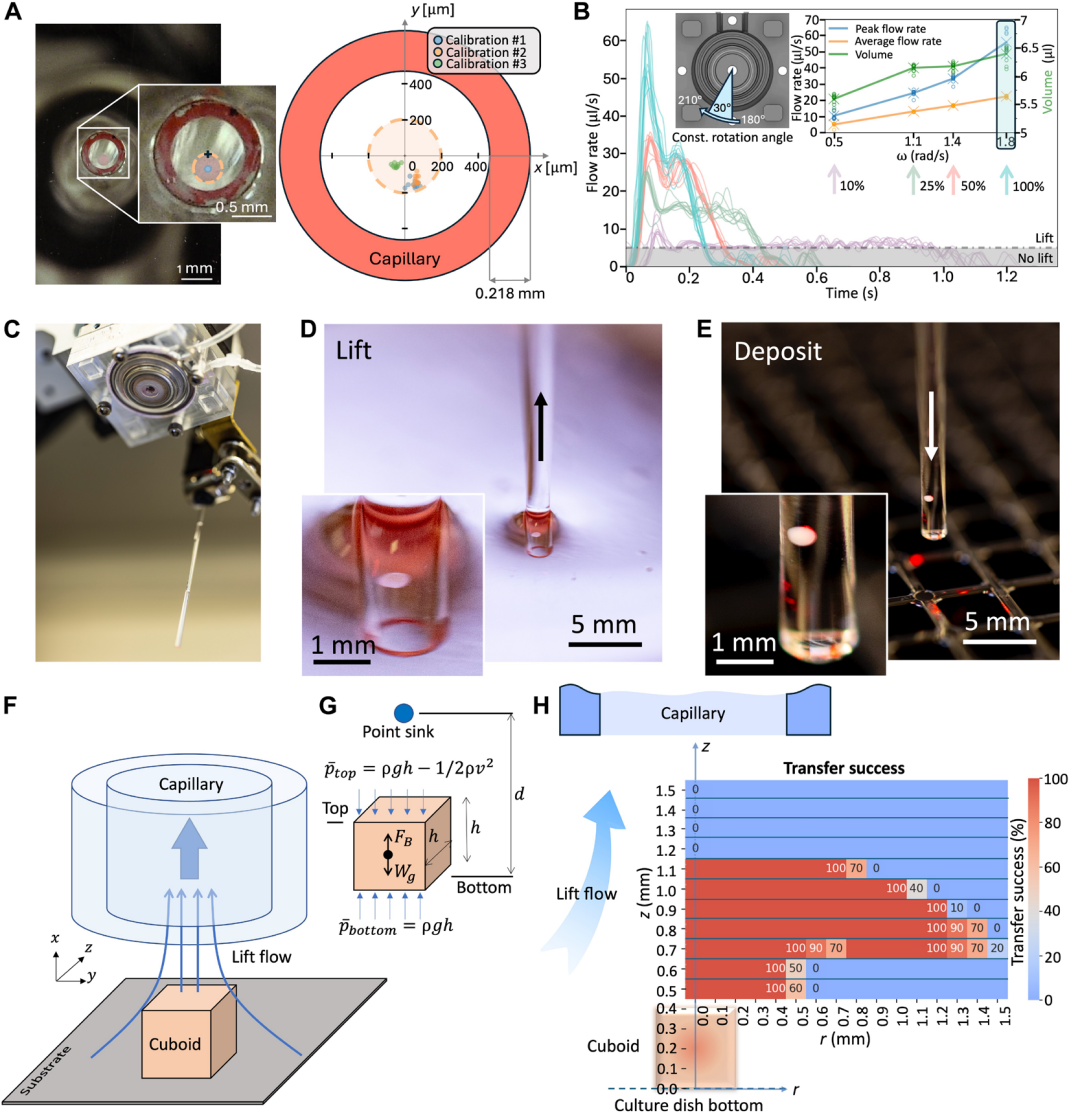
Microfluidic “lifting” of microtissues. (**A**) Accuracy and precision of pipette localization to the tissue. (Left) Images of the mouse cuboid (orange dashed circle, blue centroid) taken from underneath the dish after approaching the capillary (red painted rim). (Right) Graph depicting 30 attempts (10 attempts per calibration) to target the cuboid’s center (at the graph origin) with the capillary (shown as red torus for scale). (**B**) Plot of flow rate produced by pumping 30° rotations at four different angular speeds ω (100, 50, 25, and 10% of maximum ω, each plot consisting of 10 repeats, colored as indicated by the vertical arrows). The black dashed line indicates the lowest flow rate (~5.1 μl/s) for which there is lift. (Left inset) Pump schematic displaying the 30° rotation from 180° to 210°. (Right inset) Plot depicting the maximum/average flow rate and volume pumped for the four ω values above. The cyan shaded area indicates our operating regime. (**C**) Photograph of the pump and the capillary on approach to a culture dish. (**D**) Photograph of the microtissue being lifted into the capillary. (**E**) Photograph of the microtissue being deposited into a well. (**F**) 3D schematic setup depicting the cuboid and the lift flow entering the capillary above it. (**G**) Schematic approximation of the capillary as a point sink, along with the main model parameters. (**H**) Graph depicting cuboid picking success (for a fixed PY8119 mouse breast cancer cuboidal microtissue) as a function of radial distance *r* from the cuboid’s center and the height *z* of the capillary above the culture dish bottom, with 2D schematics of cuboid (below) and capillary (above) to scale for reference.

We evaluated the parameters that affect microtissue picking by our robotic platform. Using a high-resolution camera, we determined the accuracy and precision of the localization of microtissues. We measured the precision, or the repeatability of localization, as 26 ± 3 μm, which is well below the nominal 50 μm advertised by the robot manufacturer. We measured the accuracy, or the distance between the center of the capillary (after a given move) and the intended target (the center of the microtissue), as 129 ± 23 μm (Fig. 2A and movie S1). Note that accuracy depends on the procedure for calibrating the position of the glass capillary, which is operator dependent. Although it could be improved, the accuracy of less than half the size of the microtissue size (250 or 400 μm^3^) proved to be sufficient.

To pick up live microtissues in culture medium from the culture dish surface, we used a microfluidic “lift” process (Fig. 2, B to H) powered by a custom-made rotary pump. We rapid prototyped the pump by computer numerical control (CNC) milling poly(methyl methacrylate) (PMMA) plastic (see Materials and Methods) and installed it directly below the robot’s head. The head’s stepper motor aligns with and powers the rotary pump (fig. S1B, movie S2, and Rotary pump in Supplementary Text). The rotary pump can thus be easily programmed via Python. We used a rollerless eccentric rotary pump design to avoid pulsatile flow delivery. The pump, which only pumps air, can rotate 360° (or a fraction thereof) either backward to generate suction or forward to generate positive pressure. The pump is connected to a glass capillary [VWR International; nominal inside diameter (ID) of 0.94 mm and outer diameter (OD) of 1.37 mm]. A backward flow Q generates microfluidic “lift” that picks a microtissue off a surface (see Materials and Methods and movie S3). The fluid at rest keeps the microtissue inside the capillary during the translation of the arm (neglecting the sedimentation speed of ~500 μm/s). Forward flow dispenses the microtissue at the target location. Precise measurements of Q(t) as the pump rotates a fixed amount (30°) (fig. 2B) were obtained with a flow sensor (Sensirion SLF3S-1300F) to determine (i) the minimum Q that is needed to lift a microtissue off a surface ($Q_{\min}=$ 5.1 ± 0.3 μl/s; dashed horizontal black line in Fig. 2B, inset), (ii) the maximum Q that we can achieve (56 ± 6.1 μl/s using the highest rotational speed of the motor), and (iii) the total volume entering the capillary by integration of the Q(t) curves (~6.4 ± 0.1 μl at 56 μl/s). A large Q has the benefit of speeding up the process, and in our system, the rotary pump is consistently operated at its maximum angular velocity to achieve the highest flow rate for efficient microtissue lifting and dispensing. Figure 2 (C to E) shows a sequence of three photographs depicting the pump and capillary approach to the culture dish, the capillary suctioning a microtissue (see movie S3), and the moment when the microtissue is deposited into a well of a 384-well plate.

A theoretical fluid mechanics analysis illustrates why lifting a microtissue (in the diagram of Fig. 2, F and G, a cuboid) does not require high-precision instrumentation (e.g., a microscope or, in its default, a proximity sensor) to position the capillary in *z* near the surface. For decades, biomedical engineers have automated the manipulation of droplets (*52*), cells (*53*, *54*), colonies (*55*), embryos (*56*), spheroids (*27*), and worms (*57*). Electrostatic (*58*), magnetic (*59*), acoustic (*60*), and pneumatic soft (*61*) end-effectors have been tethered to a robotic arm to manipulate small objects. For instance, Drury and Dembo (*62*) examined the hydrodynamics of human neutrophils during micropipette aspiration, and Kumagai and Fuchiwaki (*63*) developed a capillary dispenser that could pick-and-place objects. However, to the best of our knowledge, the physical process of how microscale flow can “lift” various small objects off a cell culture surface has not been described and validated quantitatively. Our process is fundamentally different than a similarly named “aspiration-assisted” bioprinting method for precise positioning of spheroids that uses a suction cup effect to pick spheroids (*64*, *65*), whereas in our process, the pipette never contacts the cuboid(s), avoiding potential mechanical damage. In our setup, the capillary mounted at the head of the robot provides the “lift” flow (Fig. 2F) that we can approximate as a point sink (Fig. 2G). For a cuboid of volume $V_{c}$ and density $\rho_{c}$, its weight is $W=\rho_{c}V_{c}g$ and the buoyant force it experiences is $F_{B}=\rho V_{c}g$, where ρ is the density of the fluid (water). Hence, the following inequality must be satisfied

$$\left({\bar{p}}_{\text{bottom}}-{\bar{p}}_{\mathrm{top}}\right)A\geq W_{g}-F_{B}$$ (1)

where ${\bar{p}}_{\text{bottom}}$ and ${\bar{p}}_{\mathrm{top}}$ are the average fluid pressures at the bottom and top surfaces of the cuboid, respectively, and *A* ~ (400 μm)^2^ is the area of each of the faces of the cuboid. Applying Bernoulli’s equation, ${\bar{p}}_{\text{bottom}}=\rho \mathrm{gh}$ and ${\bar{p}}_{\mathrm{top}}=\rho \mathrm{gh}-1/2\rho v^{2}$, where v is the average flow velocity on top of the cuboid, Eq. 1 can be rewritten as

$$v\geq \sqrt{\frac{2\left(\rho_{c}-\rho \right)V_{c}g}{\rho A}}$$ (2)

Because v has components $u_{x}$, $u_{y}$, and $u_{z}$, where $u_{y}$ and $u_{z}\ll u_{x}$ because the capillary is centered with the cuboid, then $v\approx u_{x}$, where $u_{x}$ can be obtained from ideal flow equations through the method of images (*66*)

$$u_{x}=\frac{Q\left(x-d\right)}{{\left[{\left(x-d\right)}^{2}+y^{2}+z^{2}\right]}^{3/2}}+\frac{Q\left(x+d\right)}{{\left[{\left(x+d\right)}^{2}+y^{2}+z^{2}\right]}^{3/2}}$$ (3)

In Eq. 3, Q is the instantaneous suction flow rate generated by the pump (as measured by our flow sensor), d is the distance between the cuboid’s bottom surface and the point sink, and x, y, and z refer to the distances from the coordinate system located at the cuboid. Thus, we can find the flow velocity on top of the cuboid by substituting x=h into Eq. 3. Assuming h≪d, we obtain $v=2\mathrm{Qh}/d^{3}$. Substituting into Eq. 2, we obtain a condition for cuboid lifting to occur

$$d\leq d_{\max}={\left[\frac{2Q^{2}h^{2}\rho A}{\left(\rho_{c}-\rho \right)V_{c}g}\right]}^{1/6}$$ (4)

where h≈ 400 μm. A similar analysis follows from assuming a spheroidal shape. Note that $d_{\max}$ is not very sensitive to changes in any of the parameters. Because $A\approx h^{2}$ and $V_{c}\approx h^{3}$, then $d_{\max}∼Q^{1/3}h^{1/6}$. Substituting our typical values [Q= 56 μl/s and $\rho_{c}=$ 1.079 g/cm^3^ for mouse tumors (*67*)], we predict that the maximum distance between the capillary and the bottom of a cuboid that allows for lifting the cuboid is $d_{\max}\approx$ 1.2 mm for a 400-μm cuboid and $d_{\max}\approx$ 1.1 mm for a 250-μm cuboid.

Measurements of picking success as a function of *z* for 400-μm cuboids (Fig. 2H and fig. S4) validate this analysis. For these experiments, we used fixed PY8119 mouse breast cancer spheroidal cuboids. In agreement with our simplified point sink model, we find that the capillary needs to be equal or closer than ~1.1 mm above the surface to lift a 400-μm mouse tumor cuboid. We note that the model assumes that Q is applied as a step function, but the peak shape of Q could help lift the cuboid as soon as it surpasses $Q_{\min}\approx$ 5.1 μl/s (Fig. 2B), making the effective value of $d_{\max}$ larger. In sum, the microfluidic lift effect occurs for a wide substrate-to-capillary distance range of 0.4 to 1.1 mm (0 to 700 μm above a 400-μm cuboid), which, in practice, makes a microscope or a proximity sensor unnecessary.

### Error correction

The robotic platform incorporates software-based checks and diagnostics that prevent and correct errors in cuboid manipulation. Two main types of error can occur: an empty well or a well filled with two microtissues. To prevent the possibility of lifting two or more adjacent microtissues and determine the minimum empty distance surrounding a target cuboid, we measured the sensitivity of the capillary for picking a nearby microtissue. We first measured the success of picking a 400-μm cuboid as a function of *r*, the lateral distance between the center of mass of the cuboid and the center of the capillary, for many values of *z* (Fig. 2H). The transfer success plot displayed an interesting “mushroom” profile: right above the cuboid (within 0.4 mm, or its own height), the capillary only lifted the cuboid when its center was within the margins of the cuboid. However, at heights larger than 0.6 mm above the cuboid, the lift radius *L*_R_ from the center of the cuboid increased suddenly from *L*_R_ = 0.4 mm to *L*_R_ = 1.2 mm (Fig. 2H). At the usual *z* = 0.8 mm where we placed the (end of the) capillary, the success decayed rapidly to 0% for *r* > 1.5 mm. This mushroom profile has an immediate consequence on the ability to discriminate between two adjacent cuboids: The user can either choose to hover at low capillary-to-substrate *z* heights (risking crashing of the capillary) for high selectivity or hover at higher *z* (thus losing the ability to discriminate between two adjacent cuboids) for higher safety. We decided on higher safety because not all culture dishes are equally planar, and it is straightforward to use image recognition methods to select for properly distanced microtissues. Note that both the lateral and height spread of the red “mushroom” are very similar for the cuboid and spheroid shapes (fig. S4), indicating that the lift mechanism does not depend strongly on microtissue shape. Hence, for safety, we operated at *z* = 0.8 mm and picked only microtissues that are at least 2 mm away from another microtissue (see Transfer success in Supplementary Text and fig. S4).

To prevent picking up two microtissues at once, the software scans the culture dish and measures the distance between each microtissue and its nearest neighbor, allowing the user to filter out microtissues that are critically close to another microtissue (see Computer vision in Supplementary Text and fig. S3D). However, we observed that, in the rare cases when two small microtissues are adhered to each other, they may be interpreted as a singular, normal-size microtissue that is brought into the target well together but separates into two after ejection. Moreover, if a microtissue adheres to the inner wall of the glass capillary, its failure to eject is counted as an empty well; when the next microtissue is lifted, the microtissue buildup inside the capillary can dislodge the previously stuck microtissue, resulting in a well filled with two microtissues. In human cuboid cultures, which tended to aggregate more strongly than mouse spheroidal cuboids (likely in part due to our use of serum-free, defined medium), more than half (~56 ± 6.1%) of the wells that were filled with two cuboids were also preceded by empty wells. In mouse cuboid cultures, on the other hand, wells filled with two cuboids were never preceded by empty wells, which confirms that errors can depend on tissue type or shape.

We also addressed empty wells. Most empty wells tended to be caused by the robot’s physical inability to pick up certain microtissues, e.g., because the microtissues stick to the bottom of the culture dish, or conversely, they were not sufficiently attached during the capillary’s approach and tended to float. Because empty wells can be corrected with a second attempt, to rectify empty well errors, we used a corrective algorithm consisting of comparing images taken before and after the picking attempt (see Computer vision in Supplementary Text). If the microtissue is still in the culture dish in the same position where it was before picking, the robot returns the (microtissue-free) liquid back into the culture dish and tries again. Because the liquid ejection modifies the original distribution of microtissues at that particular location, the robot resets and attempts to pick anew. Note that our platform could not pick occasional, rare microtissues that float (e.g., those that have a high fat tissue content or have attached air bubbles) because they can easily change their position due to liquid movement after the pickup attempt. Likewise, occasional bubbles present in the culture dish can cause an empty well, but preventive measures in the software mainly take care of them (see Computer vision in Supplementary Text and fig. S3D).

To obtain statistics on the success rates of microtissue transfer by the robotic platform, we filled eight 384-well plates with microtissues from different batches: Two well plates were filled with Py8119 mouse tumor cuboids, four well plates were filled with two CRC) liver metastasis human tumor cuboids, and two well plates were filled with two intrahepatic cholangiocarcinoma (ICC) human tumor cuboids. We defined success as a well filled with a single microtissue. For mouse cuboids, we observed that 98.4 ± 0.2% of the transfer attempts were successful, 0.5 ± 0.0% were empty wells, and 1 ± 0.2% were two cuboids instead of one. Human CRC and ICC cuboids were somewhat more difficult to lift and dispense (appearing “stickier”), resulting in an average compound success rate of ~92.8 ± 1.6%, 4.1 ± 0.9% with empty wells, and 3.2 ± 0.8% with doubles.

### Selecting microtissue size and transferring multiple microtissues at once

**Fig. 3.**
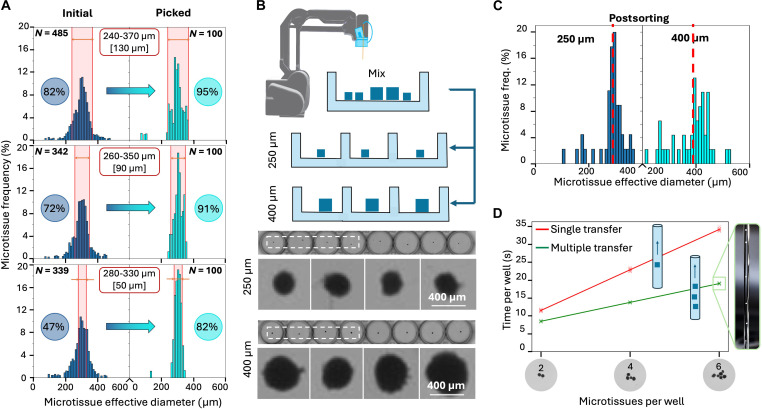
Robotic size sorting of microtissues. (**A**) Three consecutive experiments for size selection of fixed Py8119 mouse breast cancer cuboids with a decreasing picking size range from top to bottom: 130, 90, and 50 μm, respectively. The few data points below 200 μm represent camera sensor noise and debris in the culture dish. (**B**) Robotic sorting of a mixture of 250- and 400-μm-diameter live Py8119 cuboids into a 96-well plate. (Top) Process schematic. (Bottom) Grayscale micrographs of two rows of the 96-well plates after sorting and filling with 250- and 400-μm-diameter cuboids. (**C**) Postsorting size histograms for 250- and 400-μm-diameter cuboids. (**D**) Graph of robotic transfer duration as a function of number *N* of cuboids per well (depicted in the *x* axis with representative images), comparing the transfer of *N* cuboids one at a time (red line) or *N* cuboids at once (green line). (Right) Photo of six cuboids loaded into the capillary.

Microtissue size can be a confounder in studies such as cytokine secretion (the number of cells in the microtissue affects the secretion readout) and hypoxia-induced cell state (the microtissue size determines the oxygen diffusion path length, and low oxygen can lead to growth arrest or even death). The image resolution and size determination algorithm are accurate to within ±3.5 μm (see Computer vision in Supplementary Text). To reduce variability in size, we implemented a size sorting software feature that allowed us to pick microtissues with different narrow microtissue size ranges, followed by delivery to preprogrammed areas of a multiwell plate (Fig. 3A). These experiments demonstrated size ranges between 50 and 130 μm with fixed mouse tumor cuboids. The setup is not restricted to 400-μm-wide microtissues; a simple change in the size threshold in the software can also enable other applications, which call for the manipulation of much smaller and larger microtissues.

We demonstrated accurate size-selective sorting from a random mixture of 250- and 400-μm-diameter live Py8119 mouse breast cancer cuboids (Fig. 3B). The postsorting size distributions of the separated populations of 250- and 400-μm spheroidal-shaped cuboids revealed an average size of 288 and 391 μm, respectively (red dashed vertical bar in the Fig. 3C graph). The robot can also pick more than one cuboid (up to six) at once to save time if loading a well with multiple cuboids is desired; as shown in Fig. 3D, multicuboid transfer was 1.35×, 1.66×, and 1.8× faster for two, four, and six cuboids, respectively, than the added time of transferring each cuboid separately.

### Effects of robotic transfer on microtissue viability

**Fig. 4.**
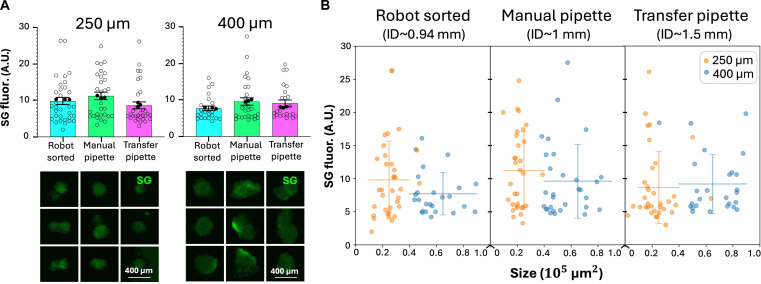
Viability analysis after robotic transfer. (**A**) Absence of viability loss in live Py8119 cuboids due to robotic transfer. (Top) Graphs of SG fluorescence (cell death) comparing three transfer methods (robotically transferred, manually transferred with a pipette, and manually transferred with a transfer pipette) for ~250- and ~400-μm cuboids. Average ± SEM, each point represents a cuboid. For 400-μm cuboids: *n* = 25–30 per condition; 250-μm cuboids: *n* = 32–35 per condition. The Kruskal-Wallis test with Dunn’s multiple comparisons test yielded no significant statistical differences for both size groups. (Bottom) Close-up micrographs of three spheroidal cuboids representative of the mean SG fluorescence (black filled points in the graph) for each condition. A.U., arbitrary units. (**B**) Comparison of cell death by SG fluorescence versus cuboid size between three transfer methods (robotically transferred, manually transferred with a pipette, and manually transferred with a transfer pipette) for ~250- and ~400-μm cuboids. Individual points and means ± SD.

To ensure that robotic transfer does not interfere with microtissue viability, we compared transfer of live Py8119 cuboids (250 and 400 μm) by robot (with ~0.94-mm ID glass capillary tube) with two standard hand-operated pipette-based methods: a P200 pipette (with tip cut to ~1-mm ID, used for precise individual cuboid transfer) and a transfer pipette (~1.5-mm ID, a gentle method also used for all bulk cuboid preparation and transfer processes). We measured cell death with the fluorescent green nuclear cell death indicator SYTOX Green (SG) 1 hour after transfer. We found no significant statistical differences between the three methods, indicating that robotic transfer causes no more notable loss of viability to the microtissues than transfer with the standard pipette-based methods (Fig. 4A). To investigate the effects of shear stress on microtissue samples, we analyzed the distribution of cell death relative to microtissue size (Fig. 4B). Shear stress is hypothesized to correlate with microtissue size as larger cuboids are positioned closer to the walls of the capillary tube. However, our analysis revealed no correlation between microtissue size and cell death; the mean and maximum cell death levels were comparable between 250- and 400-μm microtissues across all transfer methods tested. Notably, transferring microtissues using a pipette with an ID of ~1.5 mm would minimize shear stress, yet cell death measurements for this method were similar to those observed with robotic transfer and a P200 pipette. This finding suggests that shear stress does not substantially affect microtissue viability, presumably because a dead cell layer surrounding the cuboids is present immediately after microdissection, providing initial protection against flow-induced shear stress during manipulation.

### Drug tests with the robotic platform

**Fig. 5.**
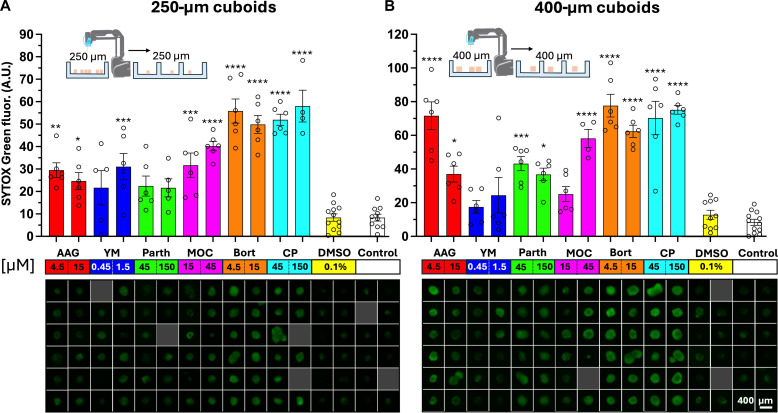
Drug testing of mouse tumor cuboids in 96-well plates. Drug treatments of 250-μm-diameter (**A**) and 400-μm-diameter (**B**) U87 mouse tumor cuboids. (Top) Bar graphs of SG (cell death) fluorescence readouts for each condition after 3 days of drug treatment. Separate batches of individually cut and segregated 250- and 400-μm cuboids were transferred into two different 96-well plates. (Bottom) Close-up micrographs of representative wells for each condition displaying green channel SG fluorescence. Each plate was treated with the following panel of drugs at two concentrations for each (drug concentration shown in μM): DMSO (vehicle control), CP (DNA/RNA synthesis inhibitor), Bort (proteasome inhibitor), MOC (HDAC inhibitor), Parth (NF-κB inhibitor), YM (E3 ligase/surviving inhibitor), and AAG (heat shock protein inhibitor). Average ± SEM, each point represents a cuboid. *n* = 4 to 6 cuboids per condition. One-way analysis of variance (ANOVA) with Tukey post hoc versus DMSO, except for CP (versus control). *P < 0.05; **P < 0.01; ***P < 0.001; ****P < 0.0001.

Mouse cuboids, with their relative homogeneity and availability, allowed us to easily test the use of the robotic platform for multiplexed drug evaluations in multiwell plates (Fig. 5). We used the robot to load U87 glioma mouse xenograft tumor spheroidal cuboids (250 and 400 μm in diameter) from two different 6-cm culture dishes into separate 96-well plates (~15 min/96-well plate, 10-μl transfer volume; see movie S4). We measured cell death with SG fluorescence at the end of the 3-day drug treatment. The drug set included cisplatin (CP; DNA/RNA synthesis inhibitor), bortezomib (Bort; proteasome inhibitor), mocetinostat [MOC; histone deacetylase (HDAC) inhibitor], parthenolide [Parth; nuclear factor κB (NF-κB) inhibitor], YM155 (YM; E3 ligase/surviving inhibitor), and tanespimycin (AAG; heat shock protein inhibitor) at two concentrations for each drug, as well as dimethyl sulfoxide (DMSO; vehicle control for all but CP) and medium alone (control). The fluorescence readouts for both 250- and 400-μm cuboid plates revealed similar strong drug responses for AAG, MOC, Bort, and CP. There were also differences. For Parth, both sizes appeared to respond but only reached significance for 400 μm. For YM, which generated highly variable responses in the cuboids, only the highest concentration with 250-μm cuboids reached statistical significance. This experiment demonstrated the suitability of the robotic platform for drug treatments using both 250- and 400-μm cuboids.

To evaluate the reproducibility of the cuboid drug treatment assay, we repeated the experiment on a different U87 mouse tumor, with 400-μm cuboids only on duplicate plates (fig. S5). We treated the cuboids with the same drug panel at similar concentrations (one low and one high). The cell death SG fluorescence readouts were similar between the two 96-well duplicate plates. Combining the results of the duplicate plates to increase our sample size, we observed statistically significant responses with AAG, Bort, Parth, and CP but not with YM or MOC, both of which showed increases but with high variability. These results were very similar to the results seen on the 400-μm cuboids from a different tumor (Fig. 5), supporting the reproducibility of the cuboid drug test.

**Fig. 6.**
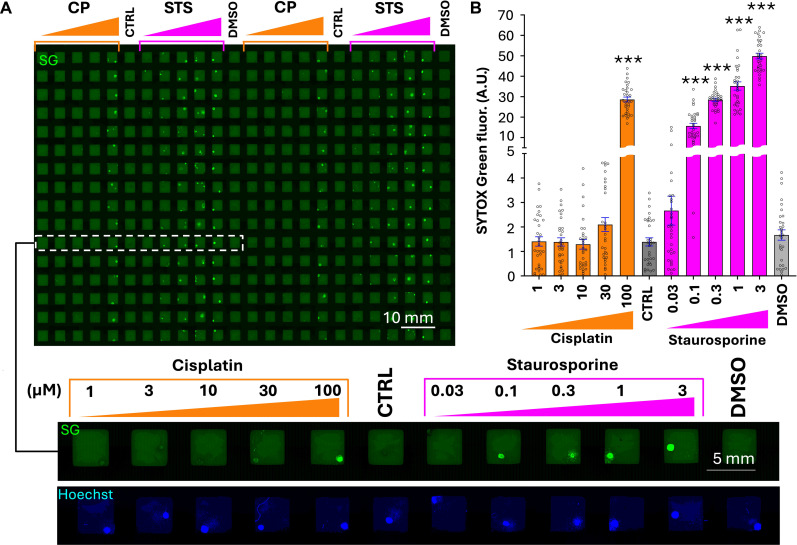
Drug testing of mouse tumor cuboids in a 384-well plate. (**A**) Py8119 mouse breast cancer spheroidal cuboids stained with SG cell death stain in a 384-well plate after 3-day drug treatment. (Top) Green channel image displaying (visually) increasing fluorescence intensity with increased drug concentration. (Bottom) Close-up micrographs of blue and green channels for one complete treatment row of CP and STS at five different concentrations each [Hoechst nuclear counterstain (blue) used for cuboid identification]. (**B**) SG fluorescence readout of 384-well plate for each condition. Average ± SEM, each point represents a cuboid. One-way ANOVA with Tukey post hoc test versus control (CTRL, no drug) for CP and DMSO (0.1%) for STS. *n* = 30 to 32 cuboids per condition. ***P < 0.001.

With the robotic platform, scaling up the drug tests to 384-well plates was straightforward, assisted by decreasing the transfer volume to ~6.4 μl. We performed a proof-of-concept two-drug dose response treatment on Py8119 mouse breast cancer cuboids in 384-well plates, in which we treated for 3 days with CP and staurosporine (STS) at five different logarithmic concentrations (Fig. 6). The use of 384-well plates allowed for ~32 cuboids per drug condition and demonstrated the robot’s ability to handle large sample sizes efficiently. In this experiment, the robot filled the plate in ~65 min with a success rate of 98.4% (378 wells of 384 filled with one cuboid); 1% (4/384) of the wells were filled with two cuboids, and 0.6% (2/384) were empty. The SG fluorescence cell death readouts for each condition revealed cell death relative to control cuboids for the two drugs, with a trend of nearly linear increasing cell death at increasing STS concentrations.

### A drug evaluation with human tumor cuboids

**Fig. 7.**
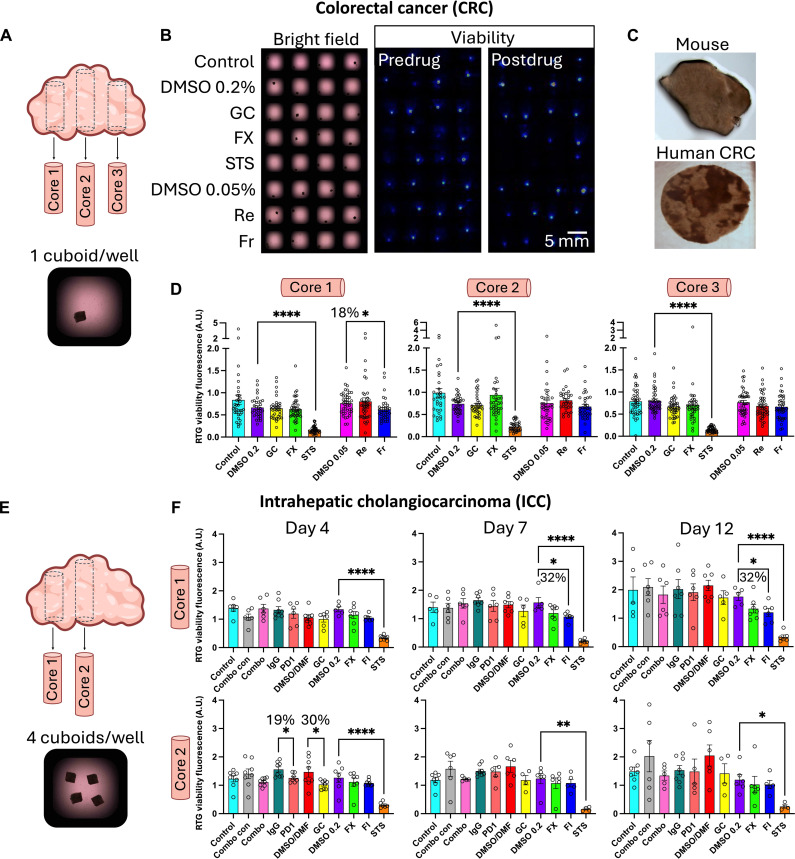
Drug testing on cuboids from two different patient tumors with clinically relevant drugs. (**A**) Cuboids were prepared from each of three core biopsies of a CRC liver metastasis tumor and distributed into 384-well plates with one cuboid per well. (**B**) Example region from the 384-well plate with cuboids from core 3. (Left) Bright-field micrograph displaying cuboid positions within wells, with each row corresponding to one condition. Conditions: DMSO vehicle control (0.2 or 0.05%), GC, FX (5-FU and oxaliplatin), STS (broad kinase inhibitor positive control), and Re and Fr (kinase inhibitors used for CRC). (Middle) Baseline viability of the same cuboids as luminescence measured with an IVIS machine. (Right) Postdrug addition viability at the sixth day. (**C**) Examples of a mouse tumor slice (homogeneous) and human tumor slice (heterogeneous). (**D**) Graphs of the drug response ratios (day 6/day 1) displayed for each core per plate. Average ± SEM, each point represents a cuboid. One-way ANOVA with Tukey post hoc. *P < 0.05; ****P < 0.0001. *n* = 30 to 43 wells per condition. (**E**) Cuboids were prepared from two cores from an ICC with four cuboids per well. (**F**) Graphs of RTG viability versus baseline for drug treatments: Combo (GC + PD-1 immunotherapy) versus combo con [0.1% DMSO, 0.1% dimethylformamide (DMF), and IgG (immunoglobulin G)]; GC versus DMSO/DMF vehicle control; FX, FI (5-FU and irinotecan), and STS versus DMSO (0.2%). Graphs of the drug response ratios for different days are displayed for each core (days 4, 7, and 12/day 1). Average ± SEM, each point represents a well with four cuboids. Four to 8 wells per condition. Student’s *t* test and one-way ANOVA, *P < 0.05; **P < 0.01; ****P < 0.0001.

The simplicity of our robotic platform is ideal for performing direct drug evaluations on human tumor cuboids in a clinical context. Toward that end, we simulated a personalized oncology drug test with clinically relevant drugs using our robotic workflow and cuboids from a patient’s CRC liver metastasis. The test took just over a week, yielding results rapidly enough, in principle, to influence treatment decisions. The patient was a 54-year-old male with recurrent CRC after previous surgery, infrared liver ablation, and chemotherapy with a combination of CP, irinotecan, leucovorin, and 5-fluorouracil (5-FU). We extracted three 6-mm core biopsies and prepared cuboids from six 400-μm-thick slices for each of the three cores (Fig. 7A). Using the robot, we filled the wells of three 384-well plates with individual cuboids, with one plate for each of the three cores, yielding an overall success rate of 91.1%. (Of the 1046 wells total for the three plates, the robot filled 953 wells with single cuboids, 41 wells with two cuboids, and 52 wells were empty.) The filled 384-well plates allowed for 30 to 43 wells per condition. Before the drug test, we measured the baseline viability after overnight culture (Fig. 7B). The baseline viability between cuboids was much more variable than in mouse, as may be expected from a heterogeneous patient tumor; as apparent in the slices used to make cuboids (Fig. 7C), some of the darker areas appeared to correspond to acellular, stromal areas (*68*). Furthermore, baseline viability differed between cores, with the highest viability from core 1, followed by core 3 and then core 2.

To test drug response, we picked four clinical drug treatments that could be considered as the next therapy for this patient. FOLFOX (FX; a combination of folinic acid, 5-FU, and oxaliplatin) and gemcitabine/CP (GC; a combination of 1 μM gemcitabine and 5 μM CP) are standard cytotoxic chemotherapies for CRC liver metastasis; regorafenib (Re; 0.5 μM) and fruquintinib (Fr; 0.5 μM) are targeted kinase inhibitors approved for CRC (*69*, *70*). In addition, STS (1 μM) was used as a positive control. DMSO vehicle controls (0.2% for cytotoxic drugs and 0.05% for kinase inhibitors) and a medium alone served as negative controls. We measured viability by RealTime-Glo (RTG) at baseline before drugs, then after 4 days for the faster acting cytotoxic drugs, and after 6 days of drug exposure as some drugs may have a slower action (Fig. 7B). RTG, unlike the single time-point fluorescent SG assay, allows multiple readings of cell death and is not affected by the high autofluorescence often found in human tissues. Cuboids from all three cores showed the expected strong response to STS (decrease of 75 ± 7%, *n* = 3 cores) and showed no statistically significant response to FX or GC (Fig. 7D). The lack of a statistically significant response to FX and GC may be in part explained by resistance from the tumor because the tumor had progressed before resection after the patient was treated with some of the compounds (5-FU in FX and CP in GC). Of note, core 1 showed a response to Fr (decrease of 18.4%, *P* < 0.05) whereas the other two cores only showed a trend that did not reach significance.

Whereas the similarity between the drug response graphs corresponding to each core speaks for the high reliability of our assay, the ability to discern small differences between cores supports that the assay can also detect differences due to heterogeneity between different regions within a patient’s TME. Furthermore, individual cuboids in separate wells also provided a measure of the spatial heterogeneity of the patient tumor within a smaller, submillimeter scale. The assay’s sensitivity and the number of cuboids required to detect a given effect are influenced by the tumor’s heterogeneity, here seen as differences between individual cuboids in separate wells. We used a post hoc power analysis to suggest the minimum sample size needed to see a certain effect with 80% power and 0.05% statistical confidence, given the variance in the values seen in this real-world test. To detect an almost complete response (e.g., the observed 75% decrease with STS), we would need to test only two cuboids per condition. On the other hand, to detect a very weak effect (e.g., the statistically significant 18% decrease with Fr in core 1), we would need to test at least 55 cuboids per condition. For an intermediate effect (e.g., a 50% decrease), we would have needed 5, 12, or 6 cuboids per condition, estimated from the variability seen for cores 1, 2, and 3, respectively.

As an approach to minimize reagent use and lower the variability in signal between wells, we conducted a drug test using multiple cuboids per well instead of one cuboid per well. We prepared cuboids from a biopsy of an untreated 56-year-old male patient with ICC, the second most common primary liver tumor. Two cores were extracted from the tumor sample (Fig. 7E). Using our robotic platform, we filled eight rows of a 384-well plate with four cuboids per well (four rows for both cores 1 and 2). The total success rate was 96.6% (176 wells filled, 1 by 1, with 704 microtissue transfer attempts in total, with 16 wells filled with three cuboids, 1 well empty, and 4 wells containing an extra cuboid). Starting at day 1, we treated each core with 11 different conditions, with eight wells per condition.

We selected five clinically relevant drug treatments and their respective controls to test drug response. The drug assay included the current first-line treatment for ICC, a combination of chemotherapy and immunotherapy, GC + checkpoint inhibitor [programmed cell death protein 1 (PD-1); 20 μg/ml]. We also used GC alone, which was the standard first-line treatment for advanced ICC for over a decade, as well as the PD-1 checkpoint inhibitor alone. In addition, we included two other drugs to treat ICC: FX and FOLFIRI (FI; a combination of folinic acid, 5-FU, and irinotecan; 1 μg/ml). Last, the assay included respective controls for the five treatments and STS positive cell death control. The same concentrations were used for FX, GC, and STS as in the CRC experiment.

To measure drug effects, we measured viability by RTG luminescence (Fig. 7F). Viability was assessed after 3, 6, and 11 days of treatment (days 4, 7, and 12). Core 1 (but not core 2) responded to FI (decrease of 32%, *P* < 0.05) at days 7 and 12, demonstrating differing drug sensitivities between different locations of the same tumor. Core 2 responded to GC at day 4 only (decrease of 30%, *P* < 0.05). On days 7 and 12, there was a trend showing a response to GC for both cores, but it did not reach significance, which may indicate that a larger sample size may be needed. As for immunotherapy, core 2 initially showed a response to the checkpoint inhibitor, PD-1, at day 4 (decrease of 18%, *P* < 0.05). However, we did not observe a response at later time points or on the other core. We also did not see a response to the combination of GC and PD-1, which would expect to be stronger than either alone. However, our viability readout could be confounded by a simultaneous decrease in signal from dying tumor cells combined with an increase in signal from expansion of activated T cells. Thus, this lack of response emphasizes the need for development of more detailed analysis methods. Overall, these results highlight the assay’s ability to capture the heterogeneity of tumor responses within the patient’s TME. Together, these findings support possible applications to personalized treatment evaluation and offer possible insights for optimizing therapeutic strategies tailored to patient tumor biology.

In sum, the robotic cuboid platform allowed us to rapidly test for differences (and similarities) in the drug response both regionally (between cores) and locally (cuboid to cuboid), highlighting the heterogeneity of the TME. The nondestructive viability assay and the standard well plate format facilitates deeper analysis of the cuboids and their TME, e.g., by cytokine secretion or omics (*51*). Hence, we believe that robot-enabled cuboid experiments could help jump-start TME-dependent research into cancer and its treatment, as well as cancer drug development.

## DISCUSSION

Our robotic platform aims to catalyze microtissue research and its applications by providing a low-cost approach to microtissue manipulation. The platform has advantages and limitations compared to existing robotic dispensers and microfluidic devices. Like other robotic approaches, the GUI does not require specialized well plates and is not limited to regular arrays for drug testing as it could be programmed to load other types of microtissue-sensing devices (*71*, *72*). The microfluidic “lifting” mechanism is universally applicable to any microtissue as long as the microtissue fits in the capillary and the pump’s flow rate is high enough to counterbalance the weight of the microtissue. Although this study was focused on microdissected tissues, we demonstrated that the computer vision and lifting mechanism work equally well for cuboidal and spheroidal microtissues (Fig. 2H and fig. S4, respectively), strongly suggesting that the platform would work for other microtissue formats such as organoids or organospheres that only differ in density or size because $d_{\max}$ is insensitive to changes in these parameters (Eq. 4), i.e., $d_{\max}∼{\left(\rho h\right)}^{1/6}$. Starting with a small biopsy microdissected into hundreds or thousands of intact TME microtissues, our low-cost robotic workflow can reliably sort the microtissues into standard multiwell plates in less than 1 hour and gather a large amount of functional information such as drug efficacy data from fluorescent readouts, but different microtissue arrays with other outputs are also imaginable (*71*, *72*). Because the microfluidic mechanism for “lifting” microtissues can be accurately explained by theoretical fluid mechanics analysis, and it is insensitive to key parameters, it should be generally applicable to a variety of microtissue formats. In principle, the pump’s physical and operational parameters can be modified to produce much larger flow rates and in larger capillaries than shown here if much larger microtissues needed to be lifted. Compared to pneumatic pipette dispensers, which contain expensive components, our compact robot can be built with low expertise and very cost-effective parts to fit in a standard tissue culture hood. Our proof-of-concept system lacks multipipetting and fluorescence imaging capabilities present in most robotic, microscope-based systems, but improvements in the camera setup (e.g., fluorescence imaging, now performed offline) could add further capabilities to our platform. Arguably, articulated robots such as ours have complex kinematics, so they are not very fast, but less complex and faster (but less cost-effective) gantry-type robots will likely achieve similar pick-and-place operations in less time and could incorporate multipipetting fluid dispensing, potentially paving the way for multiple 1536-well plate tests.

We show that the platform can work with a range of microtissue sizes (250 to 400 μm), shapes (cuboidal and spheroidal), and species (mouse and human). We focused our study on microdissected tumor “cuboids” with intact TME (*51*) because cancer drug evaluations suffer the most failures during the Food and Drug Administration approval process (more than 96%) (*2*), so oncology is the area that can benefit the most from improvements. Our drug evaluation workflow with human cuboids highlighted the value of the exercise by revealing the response variability and small differences between tumor cores. This “heterogeneity information” can only be revealed with a multiplexed functional test such as ours and could, in principle, be used to inform clinical decisions, e.g., with a similar assay performed on cuboids from a biopsy of a patient who has never undergone chemotherapy yet.

Compared to microfluidic devices that can trap microtissues in a multiwell format in parallel (*19*, *44*), our robotic platform only manipulates microtissues serially (thus slower) and cannot generate microenvironments [e.g., as needed for microvascular studies (*35*, *40*, *45*, *48*)]. However, in our platform, speed is traded for reliability, user-friendliness, and automation; in contrast to microfluidic channels which have a fixed design and have the risk of errors due to clogging, our robotic platform can be flexibly programmed to transfer microtissues into different layouts and has been designed to detect and correct its errors. Although the corrective and preventive measures in place do not completely prevent errors from happening, they substantially reduce the error rates. The small amount of failure rates remaining should not pose a problem for drug test analysis, as the failures can be easily discarded during imaging.

The robotic cuboid platform is of general applicability to drug testing, especially for TME-sensitive drugs such as immunotherapies. Hence, it could be used to develop advanced intact TME cancer models (*51*) and, with the help of machine learning algorithms trained by the cuboids’ viability data, to more directly evaluate therapeutics or combinations in intact TME human samples for the last stages of drug development (*51*). Although the heterogeneity (even of the baseline viability) can make the assay “noisy,” this variation in the drug responses likely reflects the differences in each cuboid, a strength of this approach. In a traditional bulk measure, a 100% response in half of the tissue gives the same instrument reading as a 50% response over the whole tissue. In our cuboid approach, a 100% response in half the cuboids looks very different from a 50% response in all the cuboids, allowing us to identify (and potentially further analyze) which part of the tissue corresponds to which response. Because our assay is nondestructive, downstream analysis of the cuboids, e.g., by RNA sequencing or proteomics, could provide further insights into the variability and underlying biological processes for cancer drug treatments (*51*). Hence, we believe that our platform could become an indispensable tool for the “democratization” of microtissue-based testing in a variety of scenarios, from drug development and disease models to personalized medicine.

## MATERIALS AND METHODS

### Fabrication and operation of the rotary pump

The pump uses a rollerless eccentric design to generate peristalsis along a 1.3-mm ID silicone tube (see Rotary pump in Supplementary Text for more details). The pump housing was CNC milled with a three-axis CNC mill (DATRON neo, Germany) on PMMA sheets of 8 mm thickness (McMaster-Carr, Elmhurst, IL). A central axle was 3D printed in polylactic acid (3D Solutech), using a Flashforge Creator Pro Fused Deposition Modeling 3D printer. The axle holds three metal Boca ball bearings of varying sizes, with the central bearing applying low friction peristalsis to the silicone tube, measuring 27-mm OD, 20-mm ID, and 4 mm in thickness. Two identical flanged bearings hold the central axle in place, measuring 18-mm OD and 12-mm ID with a 0.8-mm-thick flange of 19.5-mm OD. The flanged bearings provide the structural rigidity of the central axle to allow for low friction and controlled incremental movements using the stepper motor. The middle bearing is mounted eccentrically to the round inner PMMA casing. Four aluminum screws of 3 mm in diameter lock the pump to the stepper motor on the end-effector of the robotic arm. Tube replacement can be easily achieved by removing the screws and opening the housing. Peristalsis occurs as the eccentric ring bearing rolls around the silicone tube, pressed against the inner housing. The peristaltic movement generates fluid (in our case, air) displacement in the silicone tube, causing the liquid to be suctioned upward into a glass capillary attached to the silicone tube or dispensed downward out of the capillary, depending on the direction of rotation of the motor (see Rotary pump in Supplementary Text for more details). When the pump rotates a full circle, the pumping rate is not constant due to asymmetries in the design of the pump and due to the presence of the inlet and the outlet. The angle origin (0°) is halfway between the pump inlet and the outlet. We observed a peak in the pumping rate at a 30°-wide region (from 180° to 210°) opposed to the inlet and outlet, so we typically operate the pump in that region for cuboid lifting (see Fig. 2B, left inset). The settings for the motor’s angular speed ω are in percent of maximum speed, so we had to calibrate the pump’s volumetric output as shown in Fig. 2B. Settings of 10% (0.5 rad/s), 25% (1.1 rad/s), 50% (1.4 rad/s), and 100% (1.8 rad/s) of the maximum ω produced ~10.4 ± 2.2, 24.6 ± 1.6, 33.5 ± 1.9, and 56 ± 6.1 μl/s of peak flow rate, respectively (blue curve in the right inset in Fig. 2B). We were able to lift cuboids at 100% success with peak flow rates as low as 5.1 ± 0.3 μl/s (corresponding to a ω setting of 3%) and occasionally lifted cuboids with the pump’s ω set at 1% (but the lifting success diminished to 30%). In this work, the pump was always operated at 100% angular speed, resulting in a peak flow rate of 56 ± 6.1 μl/s and a total volume lifted of 6.4 ± 0.1 μl (cyan shaded area in the right inset in Fig. 2B).

### Cell culture

The Py8119 syngeneic mouse breast adenocarcinoma cell line [American Type Culture Collection (ATCC), CRL 3278] and U87-MG (ATCC) were grown in Dulbecco’s modified Eagle’s medium (DMEM)/F12 supplemented with 5% fetal bovine serum and 1% penicillin-streptomycin. Tissue culture reagents were obtained from Gibco, ATCC, or Fisher.

### Tumor generation for mouse model

Mice were handled in accordance with institutional guidelines and under protocols approved by the Animal Care and Use Committee at the University of Washington, Seattle and by the Fred Hutchinson Cancer Research Center. For the Py8119 mouse syngeneic tumors, we injected 1 × 10^6^ to 2 × 10^6^ cells in Matrigel (Corning) orthotopically into the mammary fat pad of >6-week-old female C57BL mice (the Jackson Laboratory). For U87-MG human glioma cells xenograft tumors, we injected 1 × 10^6^ to 2 × 10^6^ cells subcutaneously in 6- to 8-week-old male athymic nude mice (the Jackson Laboratory). Tumors were harvested at <2 cm^3^. If not used immediately, the tumor was stored at 4°C up to overnight in Belzer UW cold storage medium (Bridge to Life Ltd).

### Human tissue

Human tissue was obtained with written informed consent and treated in accordance with Institutional Review Board approved protocols at the University of Washington, Seattle. The CRC biopsy was from a 54-year-old male with recurrent CRC metastatic to the liver and peritoneum. He had previously received surgery, treatment with liver ablation, and chemotherapy with CP, irinotecan, leucovorin, and 5-FU. The ICC biopsy was from a 56-year-old male with an untreated, resectable ICC.

### Cuboid generation and culture

We generated cuboids as previously described (*46*). For clarity, here mouse tumor cuboids are termed “spheroidal cuboids” as they evolve into a round shape when cultured for a few days. We embedded tissue punches (600 μm in diameter, Harris Uni-Core) in 1 to 2% lo-melt agarose and then cut slices using a Leica VT 1200 S vibrating microtome or MZ5100 vibratome (Lafayette Instruments). We cut the slices into cuboids with a tissue chopper (McIlwain tissue chopper, Ted Pella Inc.) and then gently dissociated the cuboids with flow using a transfer pipette filled with serum-free medium (DMEM/F12). For 400-μm cuboids, we filtered them with a 750-μm filter to remove oversized pieces and next with a 300-μm filter to remove smaller pieces (Pluriselect). Tissue was handled using ice-cold DMEM/F12. After transfer of the cuboids to a 100-μm cell strainer (Corning or Falcon), we washed them twice with sterile phosphate-buffered saline (PBS) and once with medium. For the human CRC tumor, we did these washes in sterile tubes instead. For the CRC cuboids, the culture medium was Williams’ Media E (Sigma-Aldrich) supplemented with nicotinamide (12 mM), l-ascorbic acid 2-phosphate (50 mg/ml), d-(+)-glucose (5 mg/ml) from Sigma-Aldrich; sodium bicarbonate (2.5%), Hepes (20 mM), sodium pyruvate (1 mM), Glutamax (1%), and penicillin-streptomycin (0.4%) from Gibco; and ITS + Premix (1%) and human epidermal growth factor (20 ng/ml) from BD Biosciences. For the mouse cuboid experiments, the culture medium was DMEM/F12 with 5% heat-inactivated fetal bovine serum and 0.1% penicillin-streptomycin.

### Robotic platform setup

To become operational, the robotic platform needs to go through a physical setup process (platform assembly and capillary alignment) and a calibration between the camera (ELP 4 K IMX317 5-50 mm focus USB camera) and the robotic arm. The robotic arm (DOBOT MG-400) is outfitted with a laser pointer for calibration (used as a reference point), the custom rotary pump connected to a glass capillary, and a custom workspace platform for housing the culture dishes and well plates. The camera is positioned with a holder above the microtissue-containing culture dish. To increase the contrast (tumor microtissues are opaque), the culture dish is back-illuminated by a flat white light-emitting diode panel at all times. All devices that require power are plugged into a wall socket; the camera and robotic arm are connected to a PC with respective cables for data transfer. After the physical setup, a mapping between the robot’s and the camera’s system of coordinates needs to be established. The mapping is established with the assistance of a special calibration dish that is placed in the field of view of the camera. The robotic arm moves through the predefined positions located on the calibration dish, whereas the camera records the position of the laser dot in the calibration dish. Once the positions have been recorded, a coordinate transformation matrix can be calculated, which finishes the calibration process (see Computer vision in Supplementary Text and fig. S3, A and B). The capillary is aligned to the reference point of the robotic arm using a custom dish with a conical hole drilled in it (see Capillary alignment in Supplementary Text). The robotic platform is then ready for operation. To run the picking procedure, the user places a random distribution of microtissues (cuboids or spheroidal cuboids) in the culture dish. The software takes a picture of the culture dish and registers the coordinates of each microtissue’s projected (2D) center of mass. These coordinates are used to direct the capillary to particular microtissues. Before starting, the user is prompted to specify the range of microtissue sizes to pick from and parameters to ensure selection of isolated microtissues. The user selects the number of microtissues to transfer and defines the destination well plate format (or any other array of positions).

### Programming of the automatic tissue transfer procedure (GUI)

The user is required to initialize several input variables that affect the automatic tissue transfer procedure through a GUI. These variables include the picking size range, minimum distance to nearest neighbor, indexes of the wells to fill (in other words, how many cuboids to transfer), etc. The user must also choose the target type (a 96- or 384-well plate or a set of locations). The transfer procedure can then be started. Images taken by the camera are analyzed by the software and information on the location of the microtissues is given to the robot. The software analyzes the distribution of the microtissues and filters out those that do not fit the preset conditions. With activation of the rotary pump, the robotic arm suctions ~6.4 ± 0.1 μl of fluid, which lifts an automatically selected microtissue and reverses the pumping direction to deliver it to the target well location. In the unlikely event that microtissues within the culture dish do not satisfy any picking conditions, the process auto-pauses so their distribution can be reset by the user, here by manually shaking the culture dish. The robotic arm then continues the transfer procedure until all 96 or 384 transfers to the well plate have occurred, at which point the procedure will end. During the transfer process, the software can determine failed transfer attempts and then rectify them by refilling wells that were left empty unintentionally. Using this straightforward protocol, the robotic platform can fill a 384-well plate with single microtissues per well in ~40 min. Using a protocol that loads multiple cuboids at once into the capillary, the platform could potentially fill a 384-well plate with up to six cuboids per well in ~133 min.

### Drug treatment and imaging

To measure the viability of human tumor cuboids, RTG (Promega) was added at 1x or 0.5x and the baseline luminescence was read the following day by IVIS (PerkinElmer). Drug was added to the well, and luminescence was read again after incubation with drug without further addition of RTG. To measure cell death as an endpoint, SG (1/50,000; Invitrogen) or propidium iodide (0.1 mg/ml; Invitrogen) (with or without Hoechst; 16 μM, Invitrogen) were added to the well, incubated for 1 hour at 37°C, and then imaged with or without washing twice in PBS. We took bright-field images using a Canon DS126601 camera on a Nikon SMZ1000 dissecting scope and fluorescence images using a Keyence BZ-X800 microscope. Graphing and statistics were performed on GraphPad Prism, with power analysis performed on clincalc.com. Drugs were purchased at MedChemExpress except for STS (Thermo Fisher Scientific); 5-FU, oxaliplatin, irinotecan, Re, and Fr (Selleck); and mouse anti-human PD-1 antibody (BD Biosciences, 562138, clone EH12.1) and mouse IgG1 control (BD Biosciences, 554721).

### Data acquisition, processing, and visualization

We imaged the plates under blue and green filters using a Keyence BZ-X800 microscope to locate the microtissues and their respective average cell death fluorescence signal from the green or red channel image. We used either the blue channel (Hoechst) or the bright-field channel to determine the perimeter of the microtissues. We measured the average cell death channel fluorescence within each perimeter using ImageJ. We subtracted the background fluorescence from each cuboid and sorted all values in Microsoft Excel. Histograms were then created in Prism displaying the average, SEM, and individual data points for each drug condition. Mean RTG luminescence over each entire well was calculated using a custom Python script to define wells, retrieve signal strength, and calculate mean signal after background subtraction. For the CRC experiment, individual wells without any initial viability signal were removed from analysis. For the ICC experiment, wells low and high outliers were removed as follows. As determined by observation of control wells over time, we removed from analysis wells below a viability signal threshold at which control wells never increased. For days 7 and 12, we removed wells above the viability signal threshold at which we observed a subsequent drop in viability for control wells (presumably due to insufficient medium).

### Statistical analysis

GraphPad Prism 9 was used for tests of significance, which are done as indicated. Post hoc power analysis was performed using https://clincalc.com/stats/samplesize.aspx.

## Supplementary Materials

### The PDF file includes:

Supplementary Text Figs. S1 to S6 Tables S1 to S4 Legends for movies S1 to S4 Legends for data S1 and S2
