# Supplementary material for: Low-cost robotic manipulation of live microtissues for cancer drug testing
Source: Sci Adv. 2025 May 14;11(20):eads1631. doi: 10.1126/sciadv.ads1631 (PMC12077492; doi:10.1126/sciadv.ads1631)
Supplement: Supplementary file 3 — Data S1 and S2 [file sciadv.ads1631_data_s1_and_s2.zip › ads1631_data_s2.pdf]

# Rollerless eccentric peristaltic pump design drawings

Designed by Noah Gottshall

# Stepper motor platform where pump is mounted

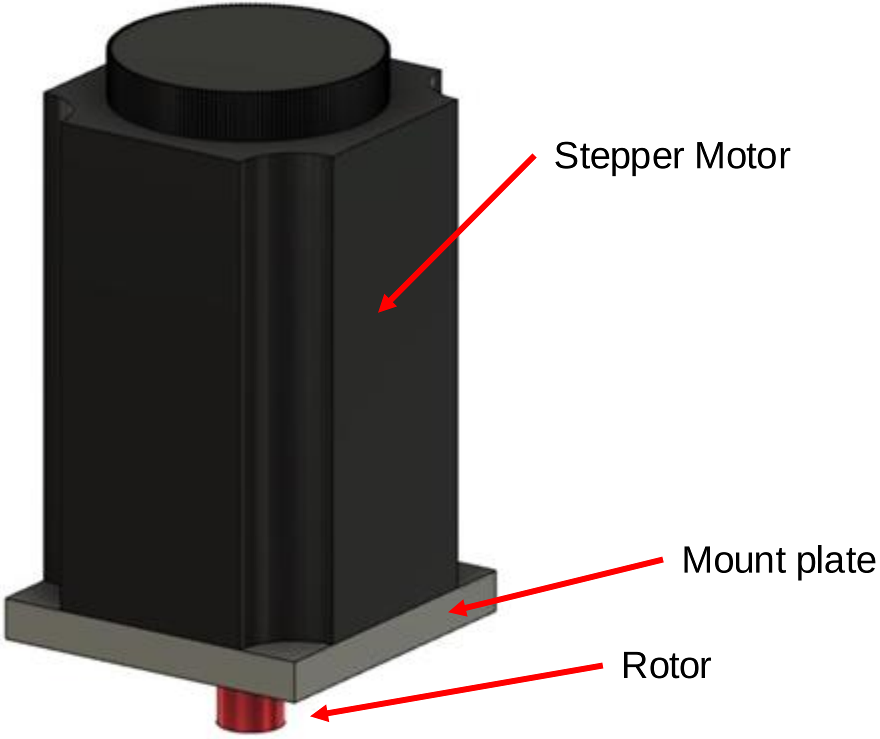


**Fig. 1: Isometric view**

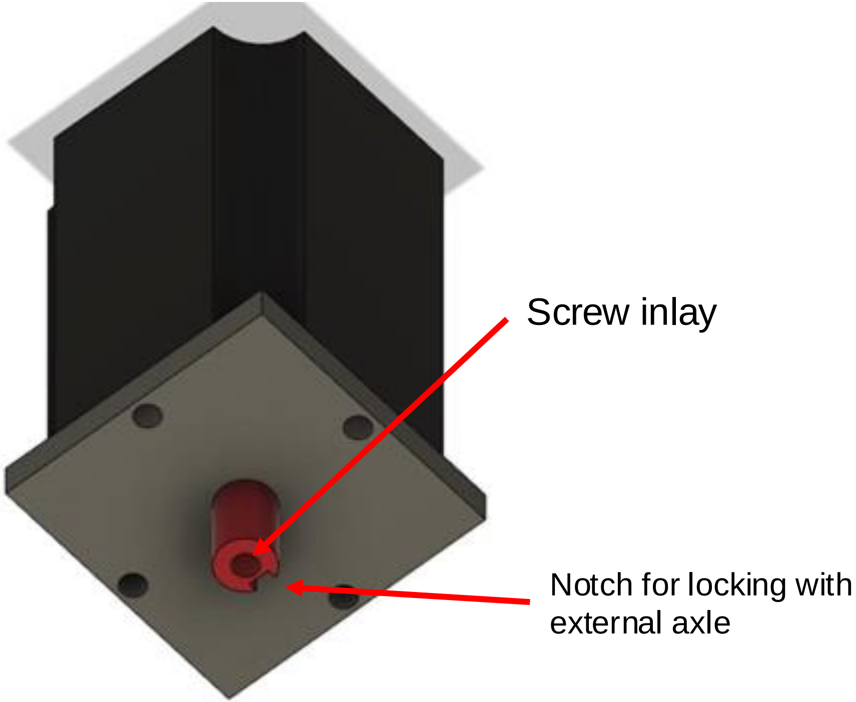


**Fig. 2: Underside view**

# Motor constrained to 1 revolution movement

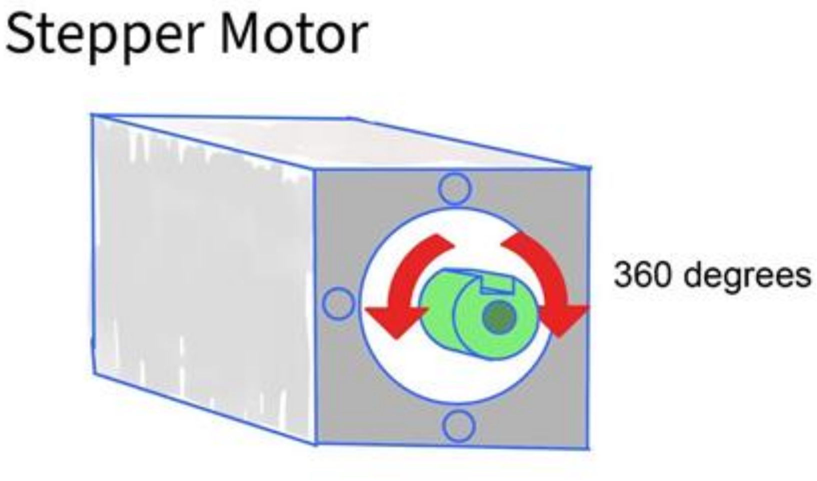


- DOBOT API limits stepper motor movement to one 360 degree rotation in either the clockwise or counter clockwise direction
- Motor cannot perform more than one full rotation

**Fig. 3: stepper motor drawing showing 360 degree restricted movement**

# Pump mounted to stepper motor platform

TOP

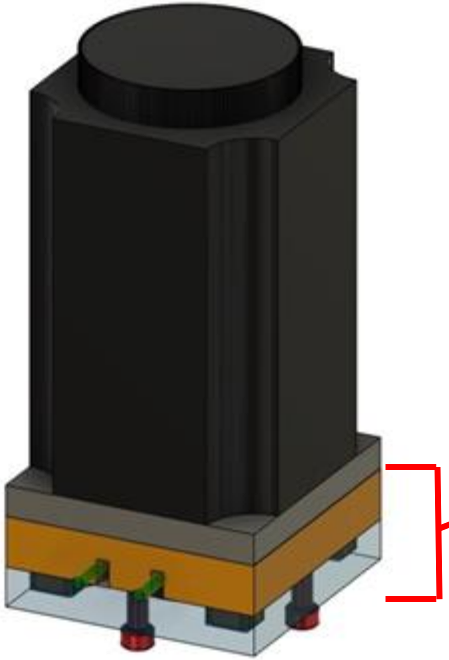

BOTTOM

Pump assembly

4 screws attach pump to motor mount

**Fig. 4: pump attached to stepper motor**

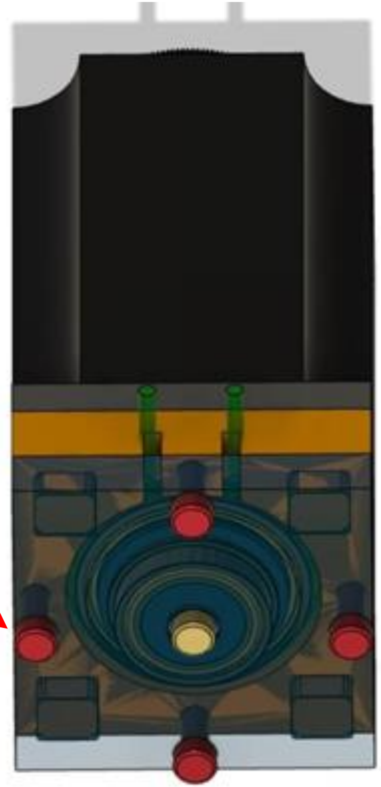

**Fig. 5: underside view**

# Pump assembly overview

TOP

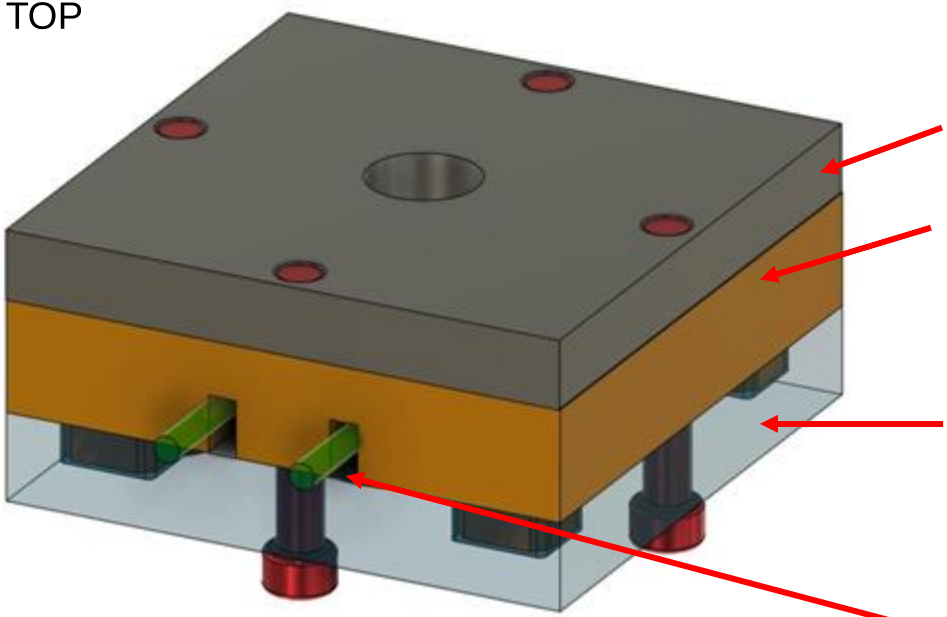

Stepper motor mount

PMMA eccentric disc housing (normally clear, colored orange for easier distinguishing from other parts)

PMMA bottom cover housing

Silicone tubing lines circular interior: Eccentric disc with ring bearing presses against tubing for peristalsis

**Fig. 6: Pump assembly**

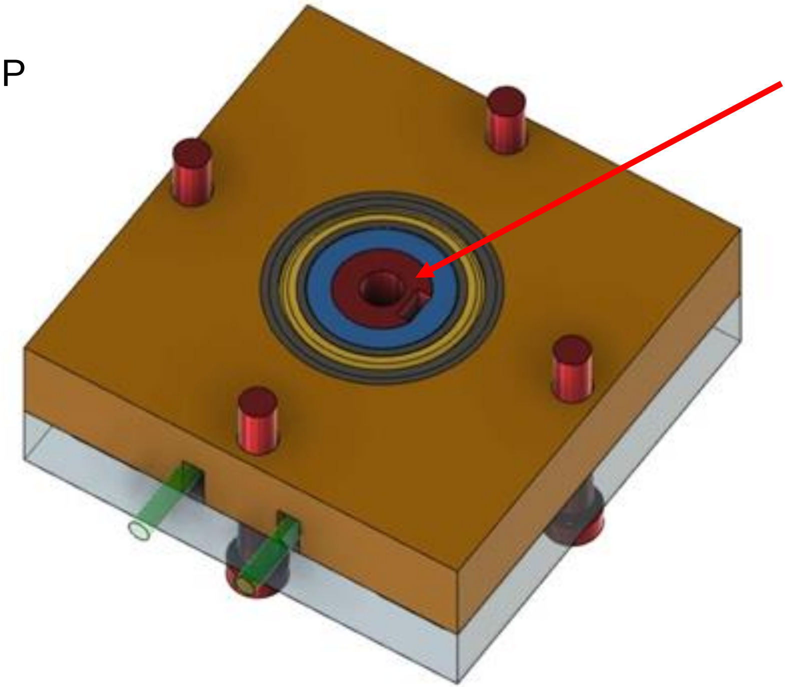


**Fig. 7: Rotor inserted into pump eccentric disc axle**

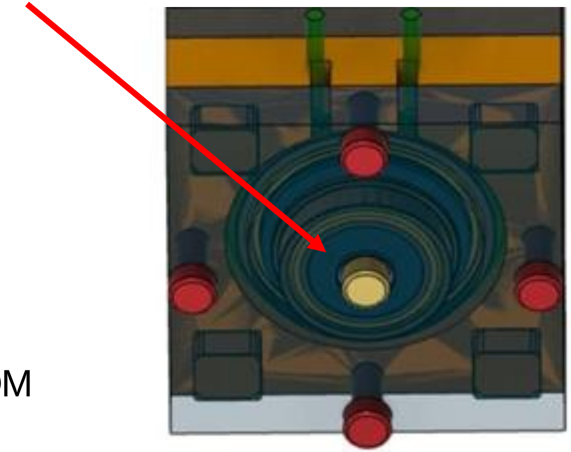


**Fig. 8: Screw inserted through eccentric disc axle and into rotor screw inlay, ensures disc locked in place with rotor**

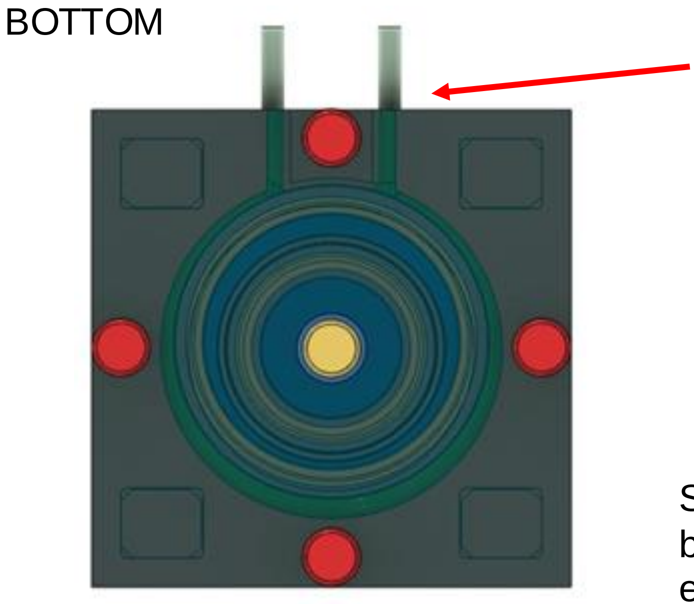


Fig. 14 Underside view

Silicone tube: runs through circular inner housing

Large ring bearing mounted on the eccentric disc axle

Second flanged ball bearing holds eccentric disc axle in place in bottom housing

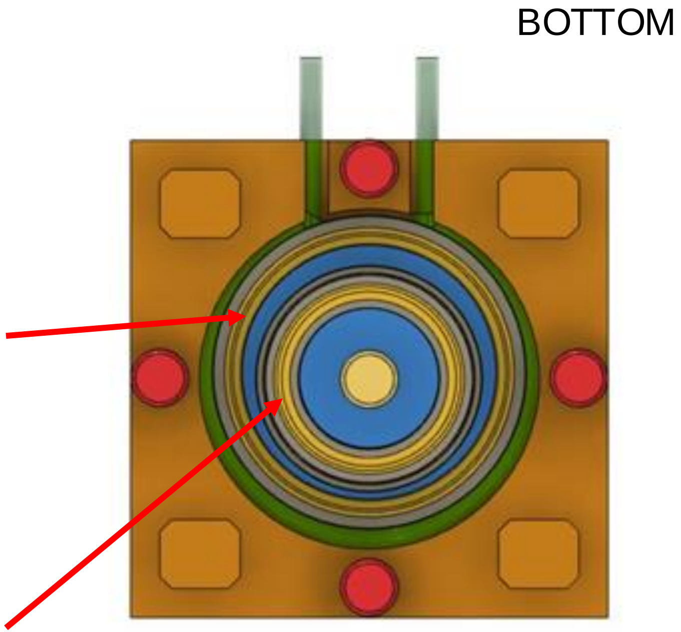


**Fig. 15 Underside view without PMMA bottom housing for better visual**

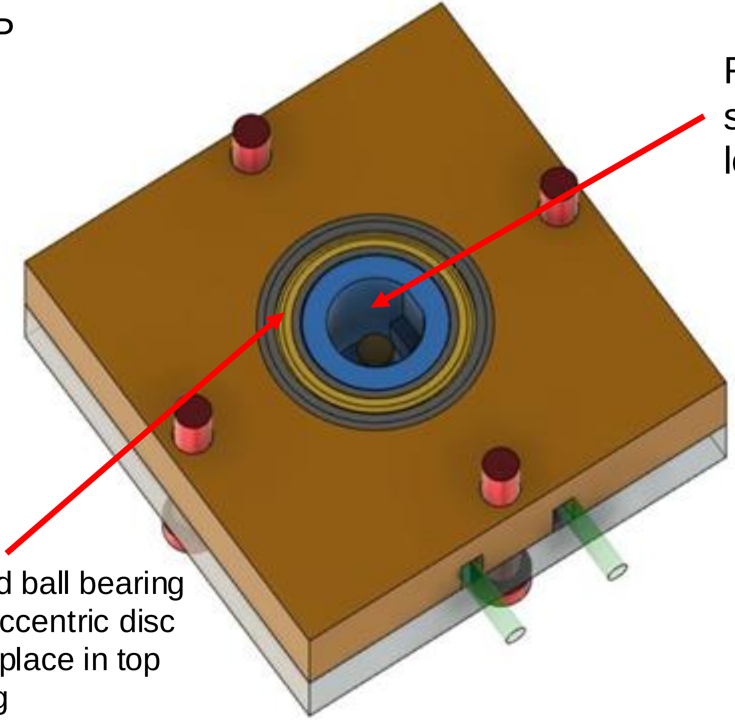


**Fig. 9 Pump showing rotor placement**

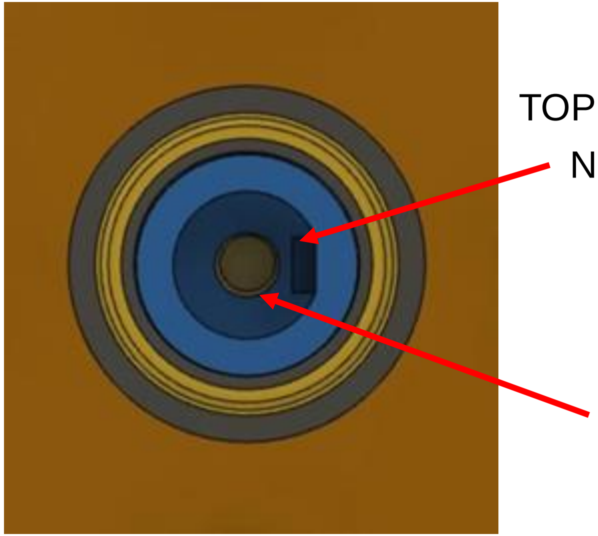


**Fig. 10 top view rotor placement**

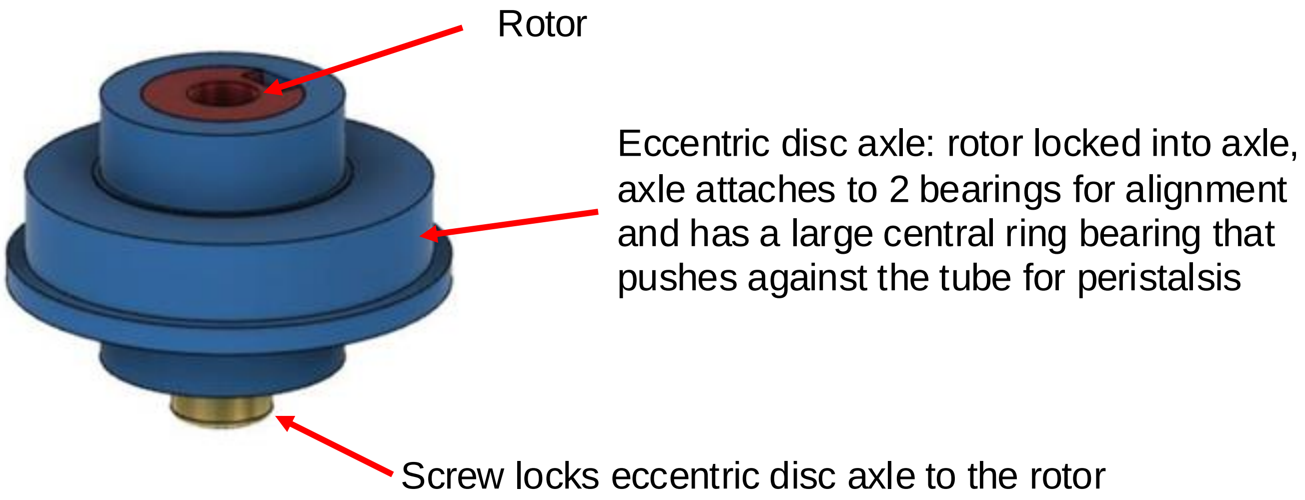


**Fig. 11: Eccentric disc axle assembly: solid 3D printed PLA axle that attaches to 2 alignment bearings and the large central ring bearing**

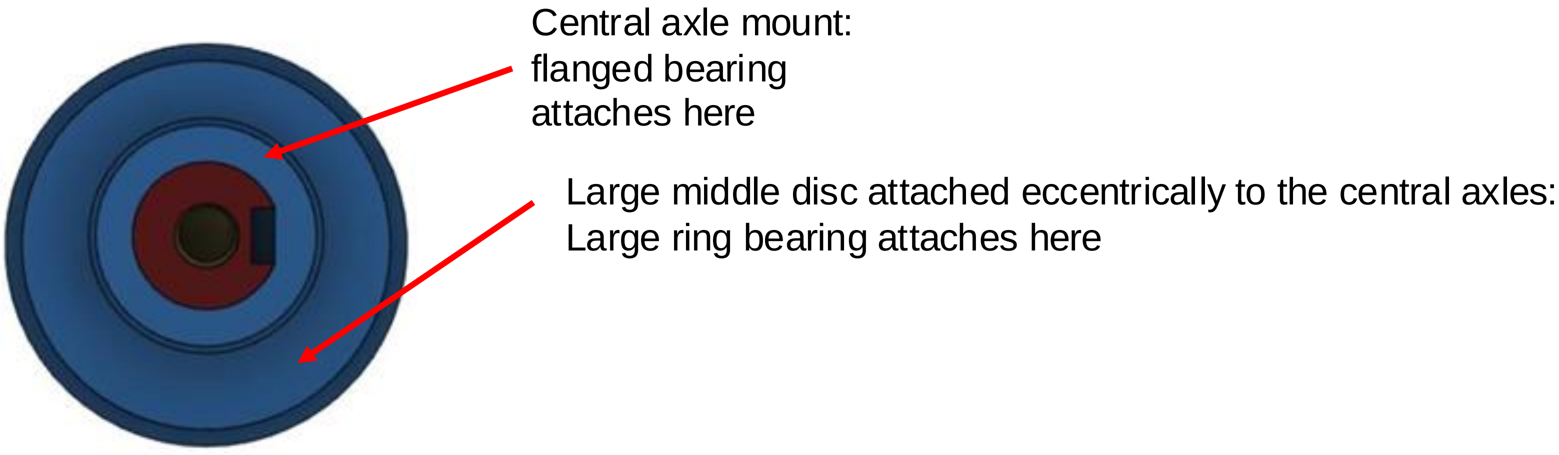


**Fig. 12: Top view of eccentric disc axle**

TOP

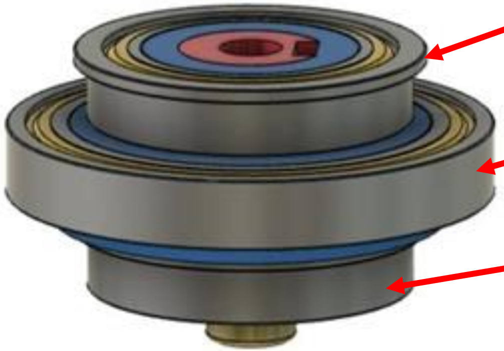

Top flanged bearing locks into upper housing for axle alignment

Large central ring bearing eccentrically mounted to axle

Bottom flanged bearing locks into lower housing for axle alignment

BOTTOM

**Fig. 13: Eccentric disc axle assembly with bearings attached: bearings attached via pressure**

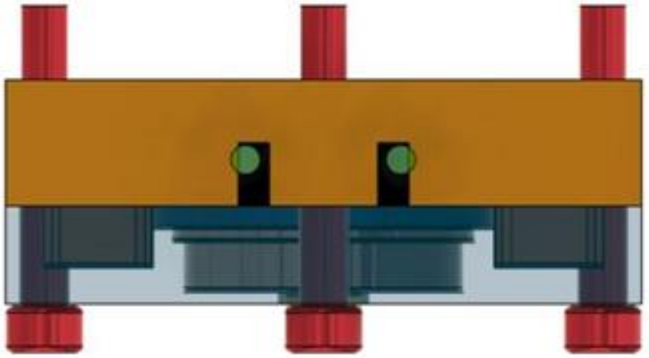


**Fig. 16: Front side view of the pump showing sandwiched layers**

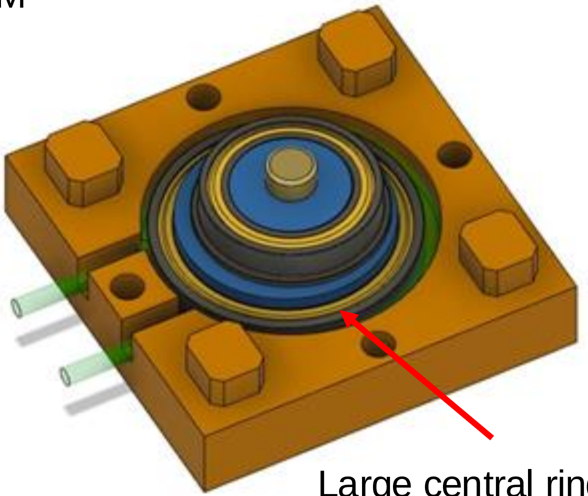


**Fig. 17: Isometric view of pump without clear PMMA bottom**

TOP

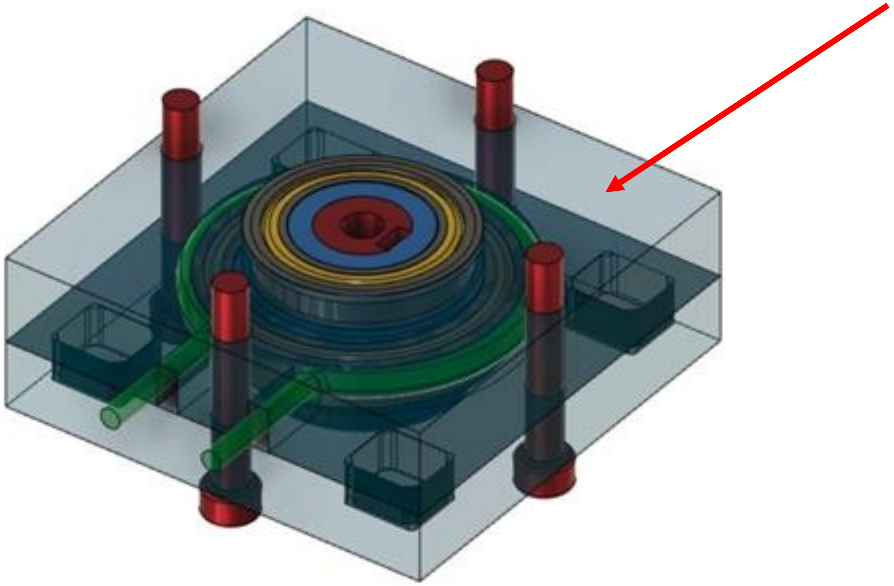

PMMA eccentric disc top housing: shown clear in this image to show what the real life pump looks like instead of using orange for clarity

**Fig. 18: Isometric view of assembled pump**

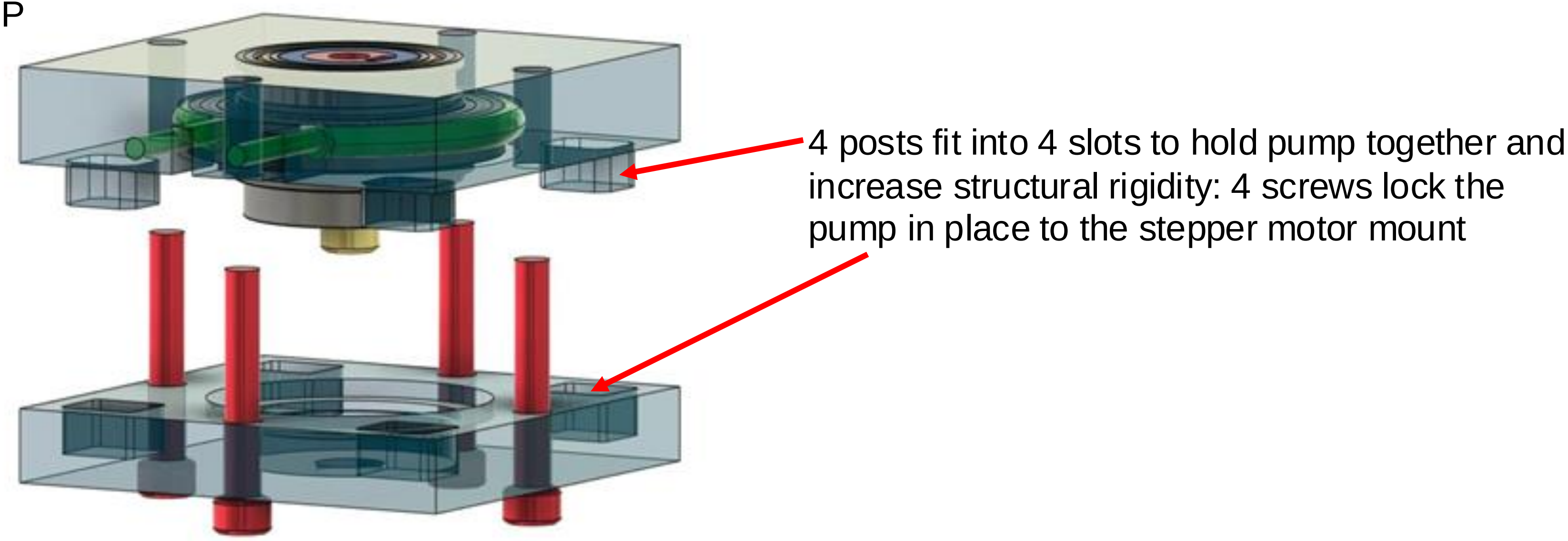


**Fig. 19: Bottom assembly removed (isometric view)**

# How the pump works

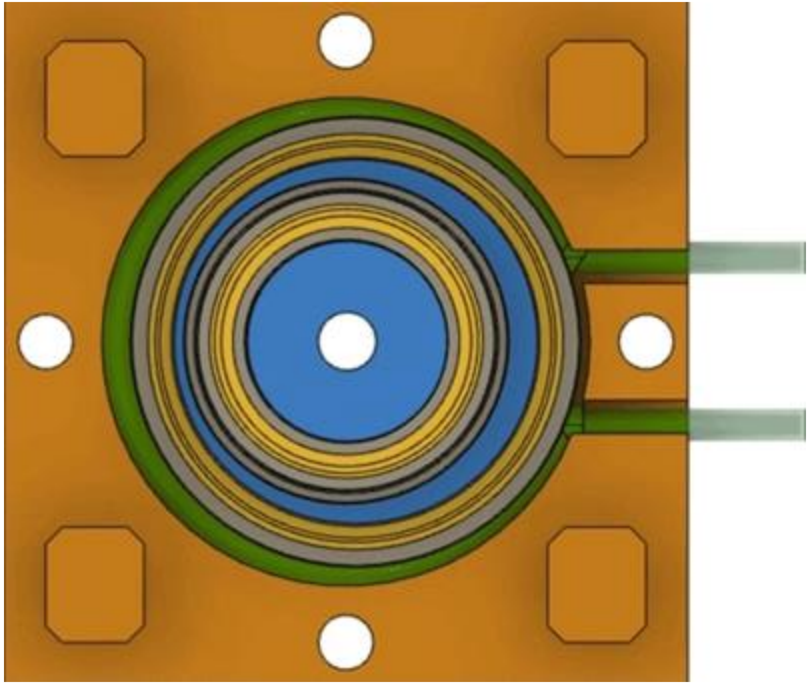

- The disc is rotated by the robot’s motor
- The ball bearing pushes against the inner tubing, causing peristalsis
- The two small flange bearings keep the eccentric disc housing aligned with the top and bottom and allow smooth rotation of the central ring bearing
- The pump rotates back and forth to pick up cuboids as shown, does not turn more than one revolution either CW or CCW
- The bearing pushes against the tubing, which causes very little friction or rubbing against the tube as it is able to roll around the tube

**Fig. 20: Animation of pump showing back and forth motion**

# Pump with unrestricted rotational movement

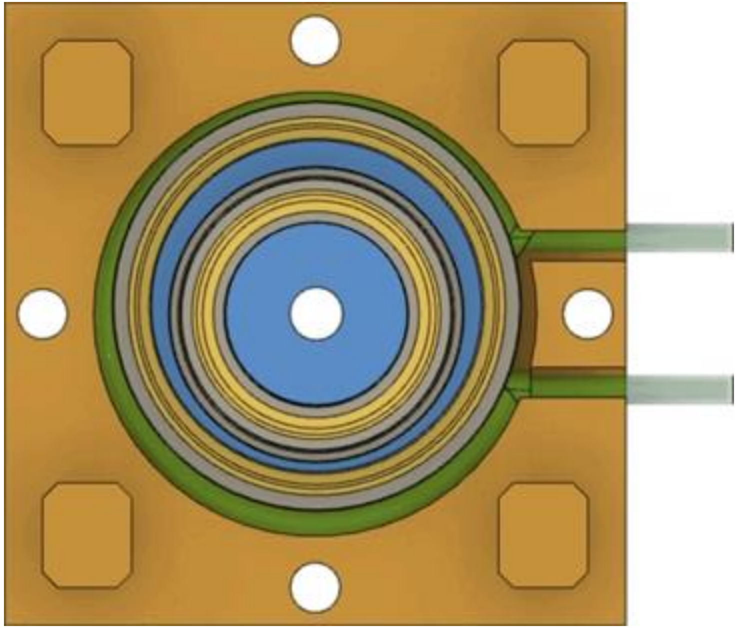

Fig. 21: Pump rotating with unrestricted movement

- The pump can technically be attached to any motor and thus can rotate more than one revolution and be used as a typical pump for other tasks

# Photos of the pump

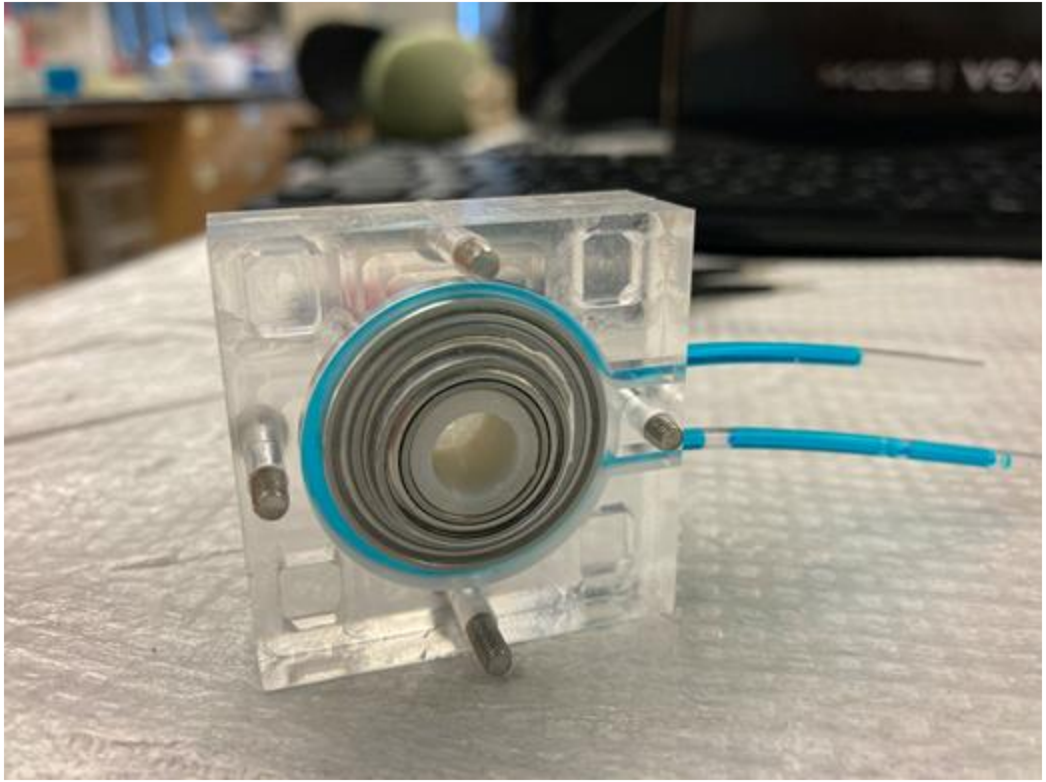

**Fig. 22: View of pump from perspective where rotor attaches to center**

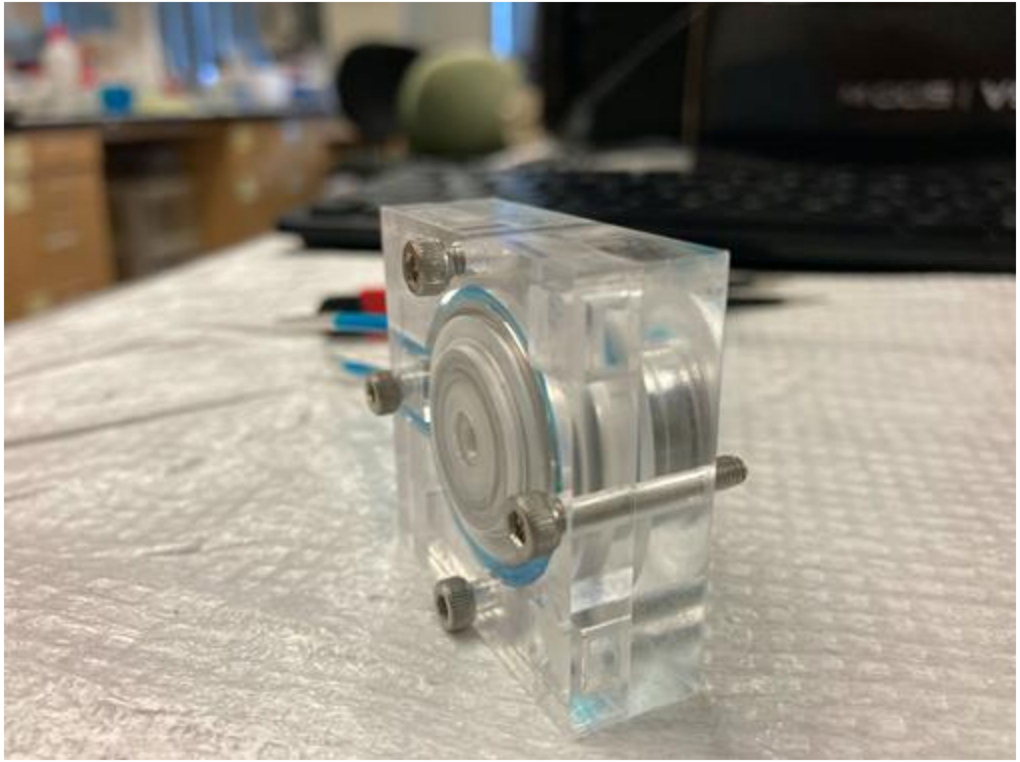

**Fig. 23: View of pump from underside perspective, shows two PMMA housing sections and screws**

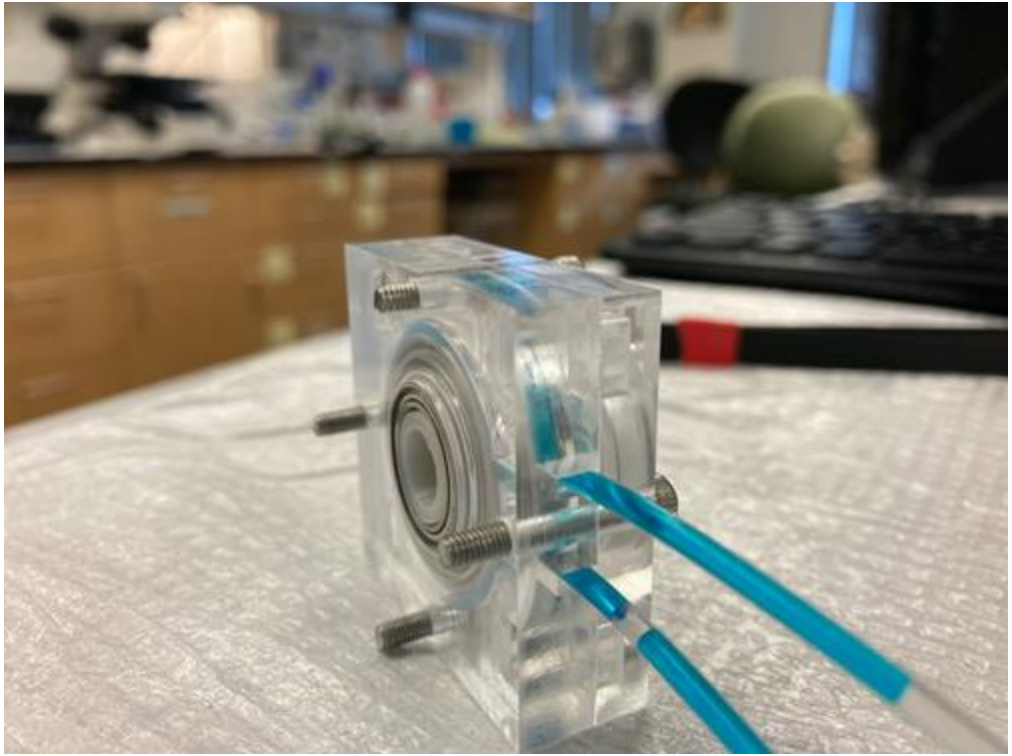

**Fig. 24: Shows how the pump is transparent**

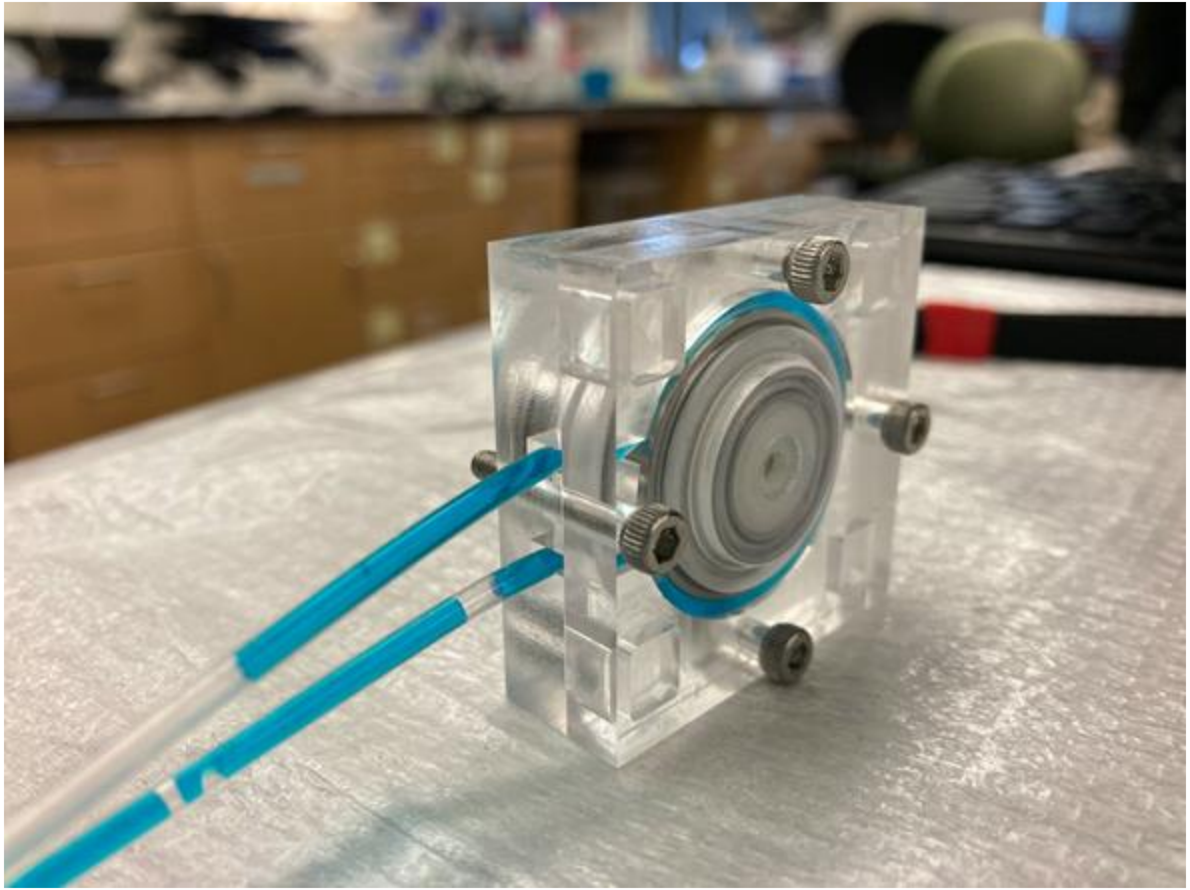

**Fig. 25 Fully constructed pump showing clear PMMA and screws used to attach to stepper motor plate. Blue fluid coloring used for visualization of tube within the pump housing**

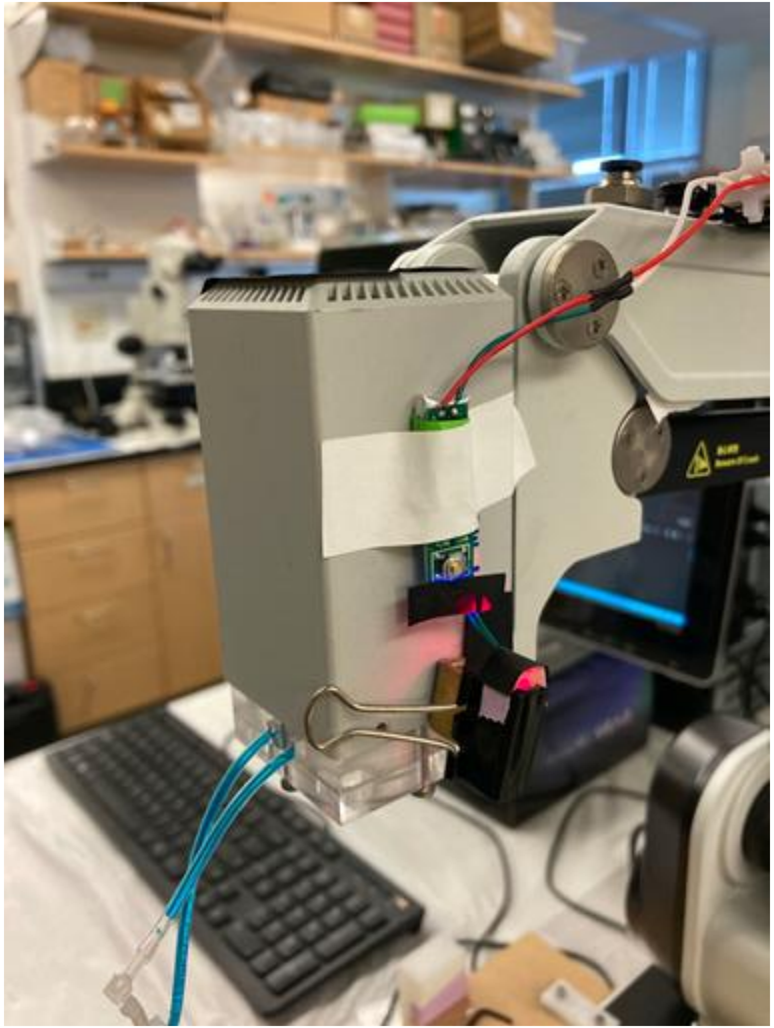

**Fig. 26: Pump mounted to DOBOT stepper motor**

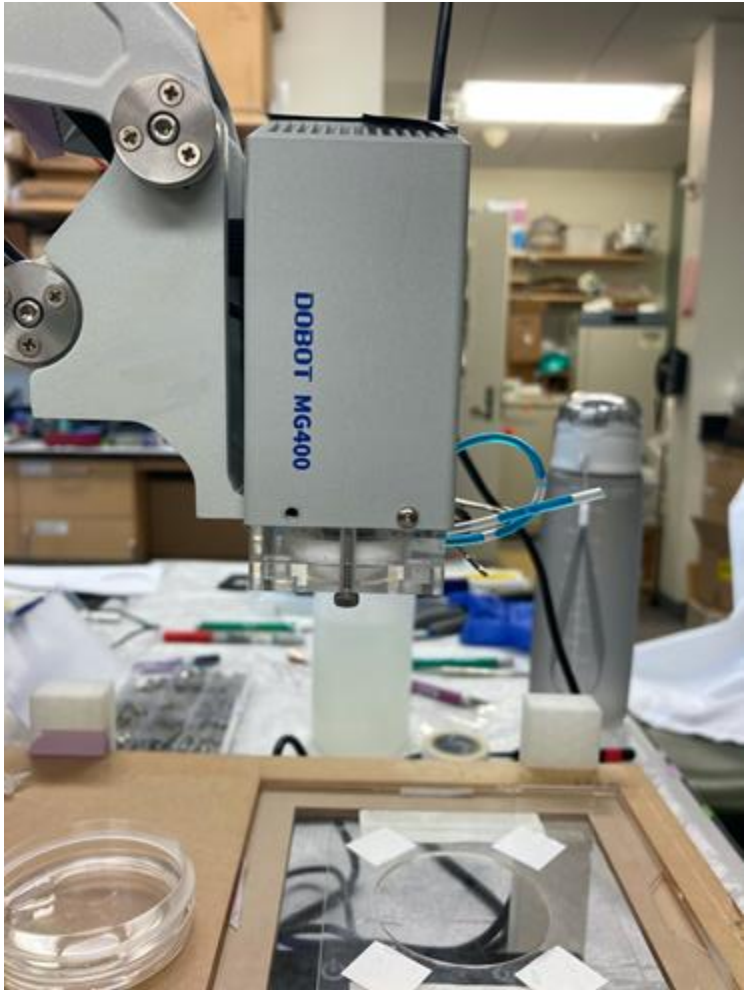


**Fig. 27: Side view of pump mounted to stepper motor**

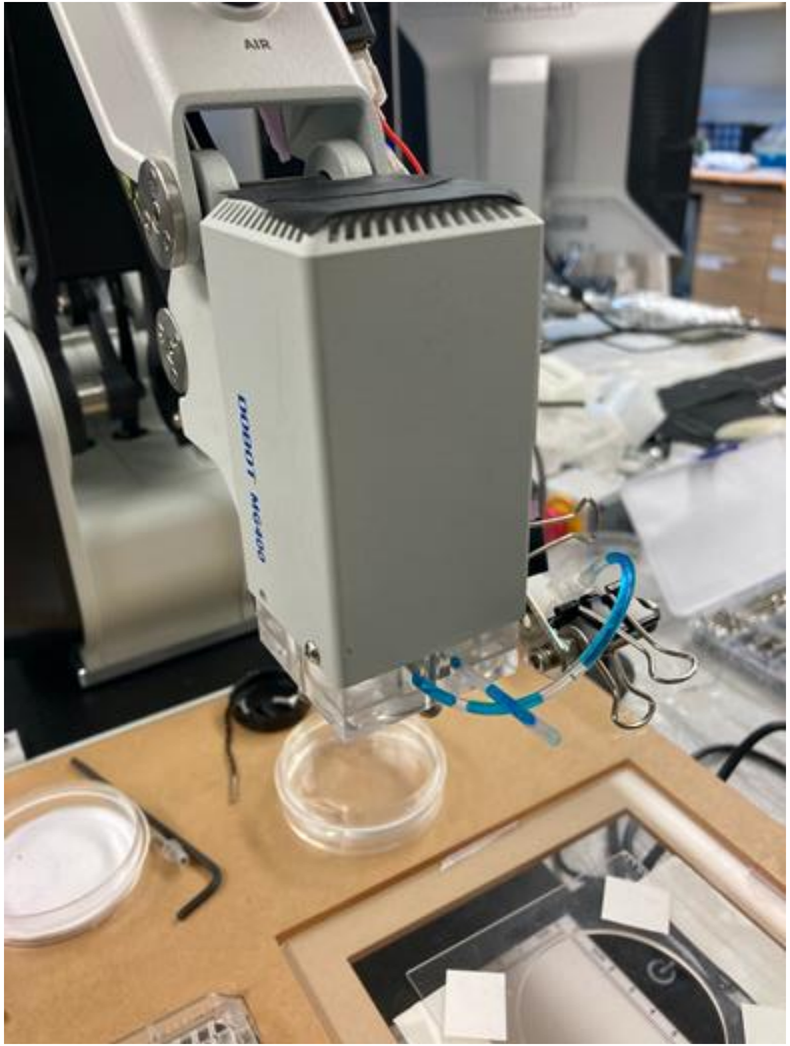


**Fig. 28 Pump mounted to DOBOT stepper motor head with 4 screws**
